# Exploration of a wide bandgap semiconducting supramolecular Mg(II)-metallohydrogel derived from an aliphatic amine: a robust resistive switching framework for brain-inspired computing

**DOI:** 10.1038/s41598-023-48936-2

**Published:** 2023-12-15

**Authors:** Kripasindhu Karmakar, Arpita Roy, Subhendu Dhibar, Shantanu Majumder, Subham Bhattacharjee, S. K. Mehebub Rahaman, Ratnakar Saha, Priyajit Chatterjee, Soumya Jyoti Ray, Bidyut Saha

**Affiliations:** 1 Colloid Chemistry Laboratory, Department of Chemistry, The University of Burdwan, Golapbag, Burdwan, West Bengal 713104 India; 2 Department of Physics, Indian Institute of Technology Patna, Patna, Bihar 801106 India; 3 Department of Chemistry, Kazi Nazrul University, Asansol, West Bengal 713303 India; 4 School of Chemical Sciences, National Institute of Science Education and Research (NISER), Bhubaneswar, Odisha 752050 India; 5 University Science Instrumentation Centre, The University of Burdwan, Golapbag, Burdwan, West Bengal 713104 India

**Keywords:** Coordination chemistry, Materials science, Soft materials, Electronics, photonics and device physics

## Abstract

A rapid metallohydrogelation strategy has been developed of magnesium(II)-ion using trimethylamine as a low molecular weight gelator in water medium at room temperature. The mechanical property of the synthesized metallohydrogel material is established through the rheological analysis. The nano-rose like morphological patterns of Mg(II)-metallohydrogel are characterized through field emission scanning electron microscopic study. The energy dispersive X-ray elemental mapping analysis confirms the primary gel forming elements of Mg(II)-metallohydrogel. The possible metallohydrogel formation strategy has been analyzed through FT-IR spectroscopic study. In this work, magnesium(II) metallohydrogel (Mg@TMA) based metal–semiconductor-metal structures have been developed and charge transport behaviour is studied. Here, it is confirmed that the magnesium(II) metallohydrogel (Mg@TMA) based resistive random access memory (RRAM) device is showing bipolar resistive switching behaviour at room temperature. We have also explored the mechanism of resistive switching behaviour using the formation (rupture) of conductive filaments between the metal electrodes. This RRAM devices exhibit excellent switching endurance over 10,000 switching cycles with a large ON/OFF ratio (~ 100). The easy fabrication techniques, robust resistive switching behaviour and stability of the present system makes these structures preferred candidate for applications in non-volatile memory design, neuromorphic computing, flexible electronics and optoelectronics etc.

## Introduction

In the modern time, scientists are involved in the evolution of functional gel materials for the benefit of mankind. Our daily life is surrounded by many forms of gels^1^. Gels are broadly studied soft materials which have 3-dimensional networks, formed by mainly two components i.e. gelator(s) and solvents(s)^1,2^. The 3-dimensional network is moulded through the immobilization of solvent molecules by the gelator(s)^2^. Development of gel is still interesting as it is the outcome of serendipity. Materials can be called as gels when it remains stable against gravitational force in the inversion vial test^2^. Amongst the classification of gels, supramolecular gels have important role in modern science for their enormous applications. Molecular self-assembly is the key tool in the formation of supramolecular gels^2–4^. Supramolecular gels are well recognized due to their enormous applications in conducting^5^, redox^6^, catalytic property^7–10^, sensing^11,12^, magnetism^13,14^, etc. The supramolecular gels are intriguing result of self-assembly of low molecular weight organic gelator(s) (LMWGs) (having the molecular mass < 3000 mg mL^−1^) and solvent molecules^15–18^. LMWGs are effective in supramolecular gel formation by immobilizing the solvent molecules through self-assembly phenomena via different types of non-covalent interactions including hydrogen bonding^15^, π⋅⋅⋅π stacking^1^, hydrophobic^2,16–18^, metal coordination^19–22^, hydrophilic^23^, electrostatic interactions^24^, van der Waals forces^15^, hydrophobic^24^, dipole–dipole interactions^23,25^ etc. Various LMWGs are reported which are responsible in supramolecular gel formation including amide^26^, tripyridine^27^, sugars^28^, carbohydrates^29,30^, alkenes^31^, aliphatic amine^32,33^, peptides^17^, steroid^34^, crown ether based gelators^35^ etc. Besides, appropriate solvents are also significant in the supramolecular gelation. LMWGs entrap suitable miscellaneous solvents like water^7,8,32,33^ methanol^36–39^, ethanol^40–42^, dimethylformamide^43–46^, dimethyl sulfoxide^47–50^, acetone^51^, acetonitrile^52–54^, dichloromethane^55^, toluene^56^, etc. to form supramolecular gel.

Recently, metallohydrogel has been found to be an expanding class of supramolecular gel chemistry which is the output of diverse non-covalent interactions along with metal–ligand coordination^57^. Supramolecular metallohydrogel is eminent for limitless applications in semiconducting^43–46^, catalytic property^7–10^, proton conductivity^58,59^, cellular encapsulation^60^, tissue engineering^61^, biomedical applications^62^, solar cell^63^, power source^64^, etc.

Until now numerous metallogels have been synthesized using different metal ions like magnesium(II)^32,33,65^, vanadium(V)^66^, manganese(II)^3,4^, iron(II/III)^57,67–69^, cobalt(II)^7,63,70,71^, nickel(II)^72,73^, copper(II)^47,74,75^, zinc(II)^76,77^, cadmium(II)^43,78^, mercury(II)^79^, europium(III)^54,80^, terbium(III)^46,54,80^, neodymium(III)^81^, to apply on various applications^2,82^. Magnesium metallogels have various applications like iodine sequestration^83^, organic dye adsorption^33^, coordination polymer^84^, thermo-responsive material^65,85^, self-healing property^32,33^, superconductor^32,83^, etc. In order to construct electrical devices based on metal–semiconductor (MS) junctions, we have introduced trimethylamine as an LMWG based supramolecular magnesium(II)-metallohydrogel, while preserving a straightforward synthesis technique at ambient temperature in water medium.

On the other hand, due to its superior memory characteristics and broad variety of applications in several fields, like switching, non-volatile memory design, neuromorphic computing etc.^86–88^, resistive random access memory (RRAM) devices^89–91^ have been the center of significant research. The operation of RRAM devices is based on resistive switching process where the resistance state of the system goes through a distinct high(low) resistance state which is guided by physical mechanisms such as vacancy migration and the trapping/separation of charge carriers etc. Due to the compatibility with CMOS architecture, its straightforward structure, excellent manufacturability, cheap price, low power consumption, fast speed, long endurance, and reliability, it is a chosen choice for next-generation memory design. While oxide materials are widely studied for RRAM design^92–97^, but scientists are looking for alternative materials to overcome the material challenges and obtain better performance. The metallohydrogel can work as an active material to design such RRAM structures^83^, which can be useful for flexible electronics design as well.

In this recent research work, we have been successful to fabricate Mg(II)-metallohydrogel (Mg@TMA) mediated metal–semiconductor (MS) junction based resistive random access memory (RRAM) devices for switching and non-volatile memory application. Our approach of using functional flexible soft gel-scaffold may pledge towards the arena of memory device based on research and technology for potential applications in neuromorphic computing^98–100^ and data driven applications like 5Getc.

Novel computing architectures are proposed to solve the von Neumann bottleneck, where the physical separation between the data processing and the memory units in conventional computers pose increasing limitations of latency and power consumption, especially for data centric computation.

The von Neumann bottleneck can be solved either by creating a 3D structure for co-integration of computing and memory elements, or by introducing whole new architecture concepts of in-memory computing, such as in-memory logic, and neuromorphic computing. These research efforts generally on novel switches, such as resistive switching memory (RRAM) which can serve as memory and computing element at the same time. Recently, in-memory computing circuits take advantage of the RRAM ability to add multiple applied pulses, which acts as a logic gate. RRAM logic gates rely on the conditional switching in one or more output RRAMs, depending on the applied voltage amplitudes and the states of input RRAM devices. RRAM device can be used as synaptic elements in brain inspired computing. The use of RRAM devices as synaptic or neuron elements, requires optimization of certain properties, such as multilevel operation, high on/off ratio, linear change of the resistance upon set and reset, and good reliability. RRAM have a large application in memory, logic operations and neural synaptic networks due to their non-volatility and nonlinearity. Our work presents a concise overview of RRAM-based logic circuits and analyses their role in memory applications. Furthermore, this work explores the possibilities of utilizing memristors for implementing logic in memristor arrays. Therefore, research on memristor-based logic circuits opens new possibilities and methodologies for designing innovative logic architectures. These memristor-based logic operations are categorized as an exploration of novel logic circuits.

**Figure 1:**
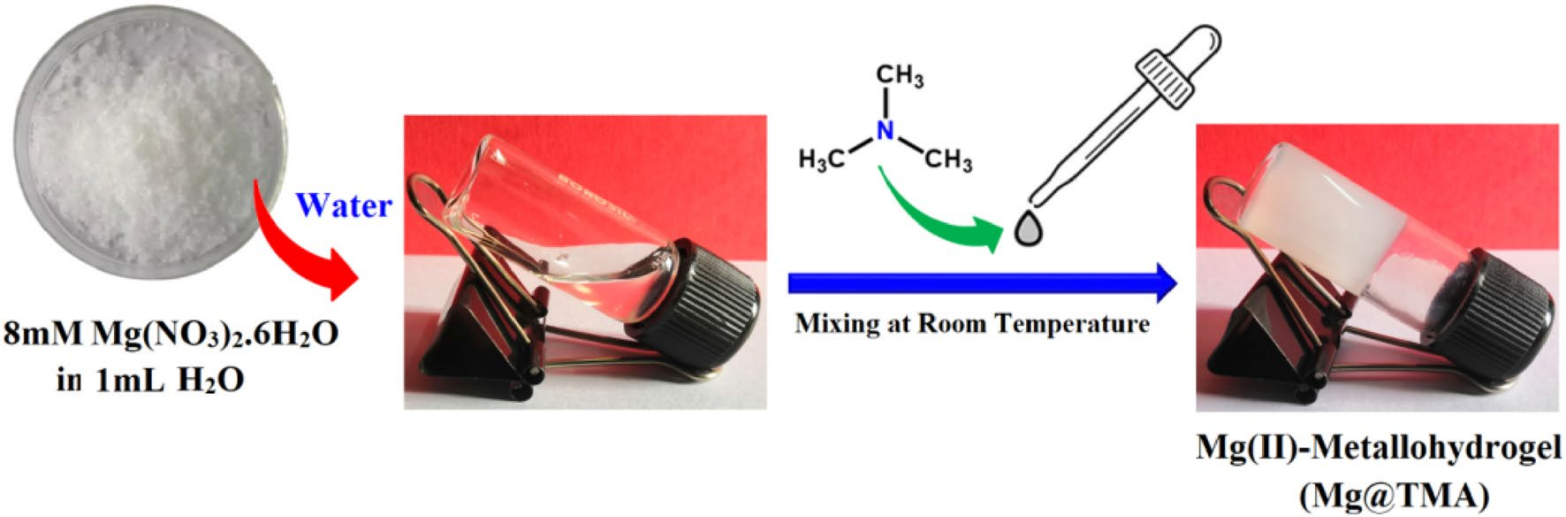
Synthetic method of Mg(II)-metallohydrogel (Mg@TMA).

**Figure 2:**
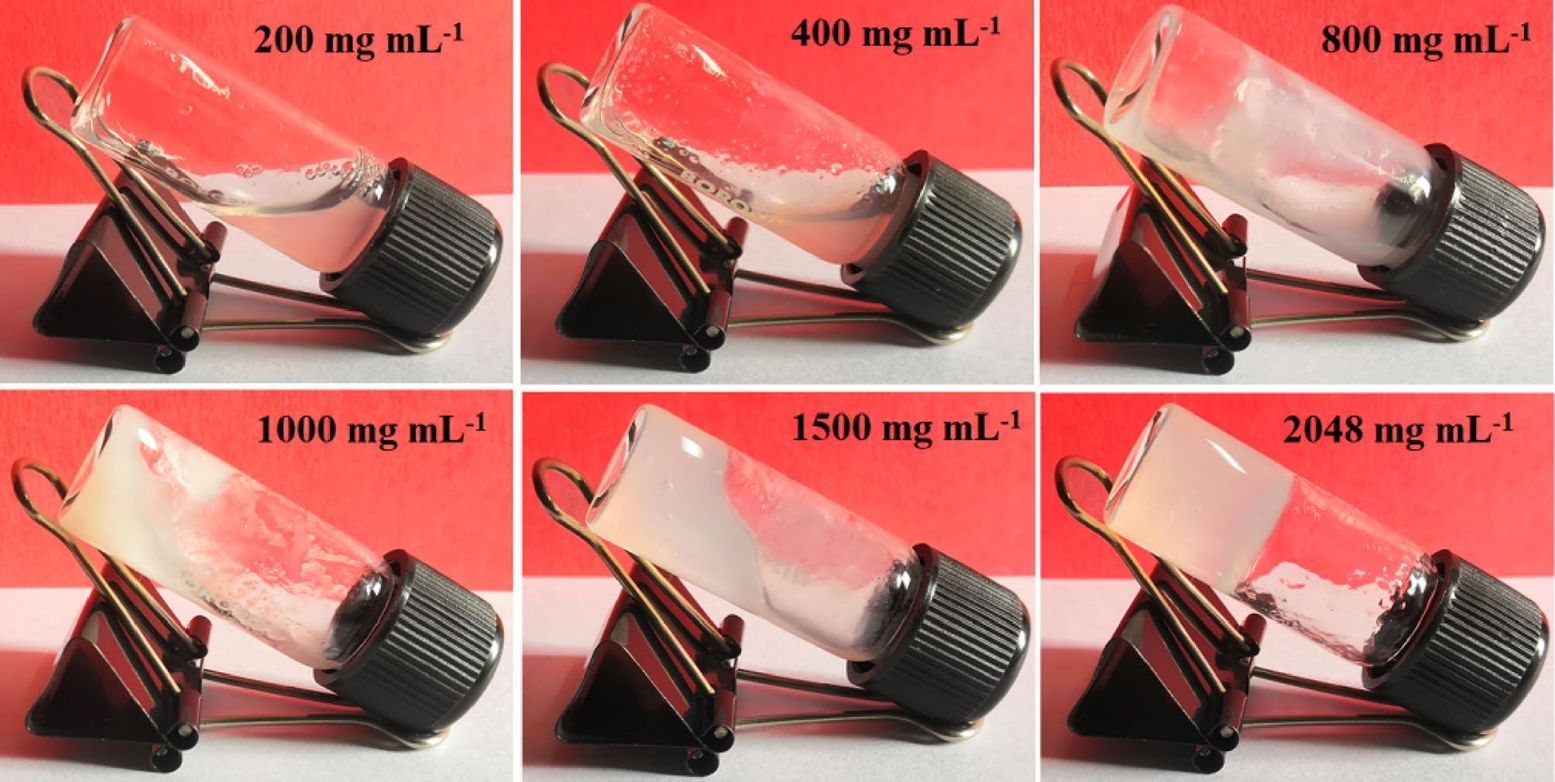
The gelation process of Mg@TMA metallohydrogel.

In this work, the white-colored magnesium(II) metallohydrogel, known as Mg@TMA, was prepared by quickly mixing approximately 1 mL of an aqueous solution containing 2.048 g (8 mmol) of Mg(NO_3_)_2_·6H_2_O and 1 mL of trimethylamine (TMA) (refer to Fig. 1). This synthesis process resulted in a stable Mg(II)-metallohydrogel that retains its white color even when stored at room temperature for several months. Figure 2 illustrates the determination of the minimum critical gel concentration (MGC) of Mg@TMA by adjusting the concentration of the metal salt within the range of 200–2048 mg mL^−1^. The MGC was found to be 2048 mg mL^−1^.

## Results and discussion

### Rheological analysis

The rheological mechanical property results from angular frequency and strain-sweep measurement tests confirmed the viscoelastic nature of the Mg@TMA metallohydrogel. This is a case where the storage modulus (G′, the energy stored in the system during the application of shear to the viscoelastic zone) is much greater than the loss modulus (G′′, the energy lost to the system during the application of oscillatory stress).

**Figure 3:**
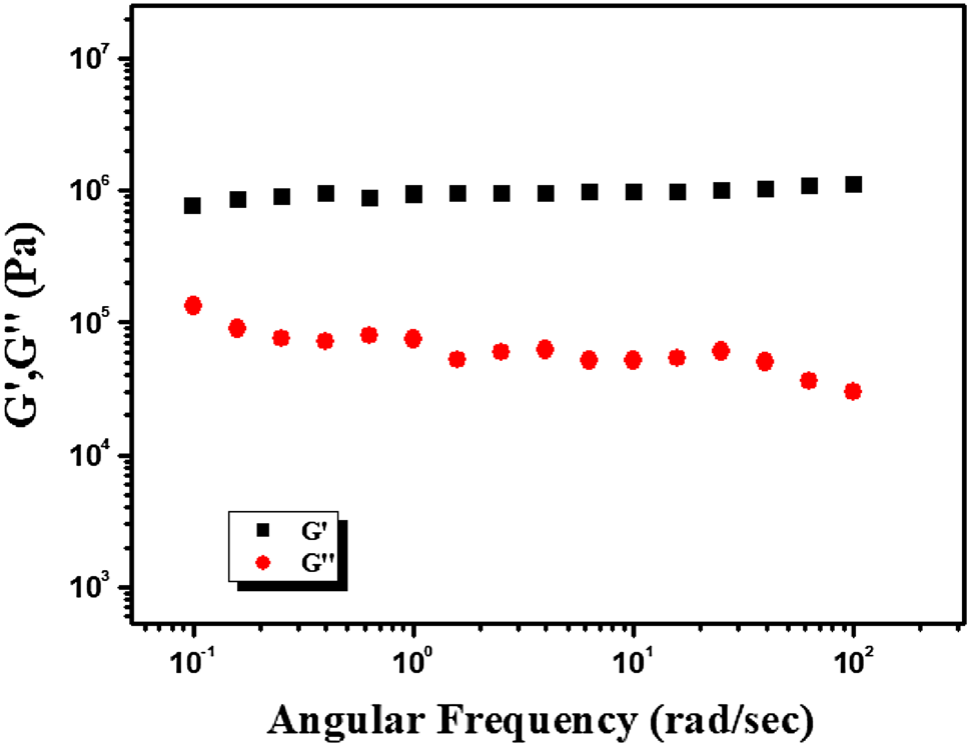
Angular frequency-dependent storage modulus (*G′*) and loss modulus (*G″*) of the Mg@TMA metallohydrogel.

**Figure 4:**
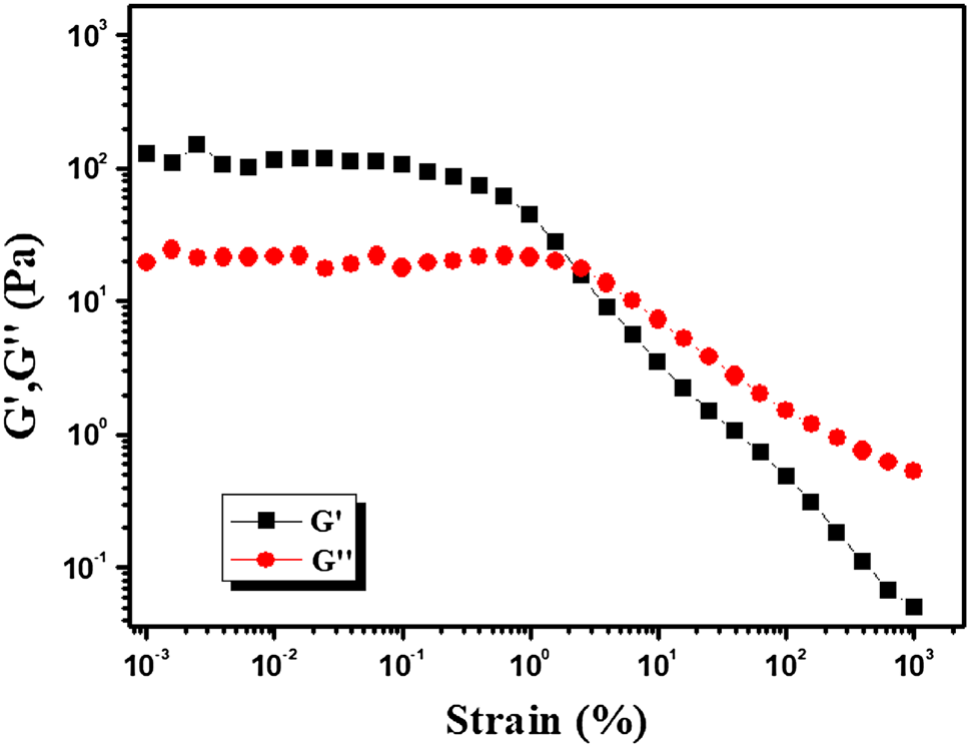
Strain-sweep measurements of Mg@TMA metallohydrogel.

Mg@TMA metallohydrogel’s gel nature is verified at a fixed concentration of Mg(NO_3_)_2_.6H_2_O and trimethylamine (i.e. MGC = 2048 mg/mL) (Fig. 3). Tolerance limits of Mg@TMA metallohydrogel are set by its average storage modulus (G′), which is much greater than its loss modulus (G′′) (Fig. 3). Moreover, strain-sweep measurements of Mg@TMA metallohydrogel were captured (Fig. 4). The Mg@TMA metallohydrogel strain-sweep measurement was carried out at a fixed frequency of 6.283 rad/s (Fig. 4). The critical strain (minimum strain for gel breaking) of Mg@TMA metallohydrogel is shown by the strain-sweep measurement as G′ crossing G′′ at 2.2247% strain (Fig. 4).

### Microstructural study of Mg@TMA metallohydrogel

**Figure 5:**
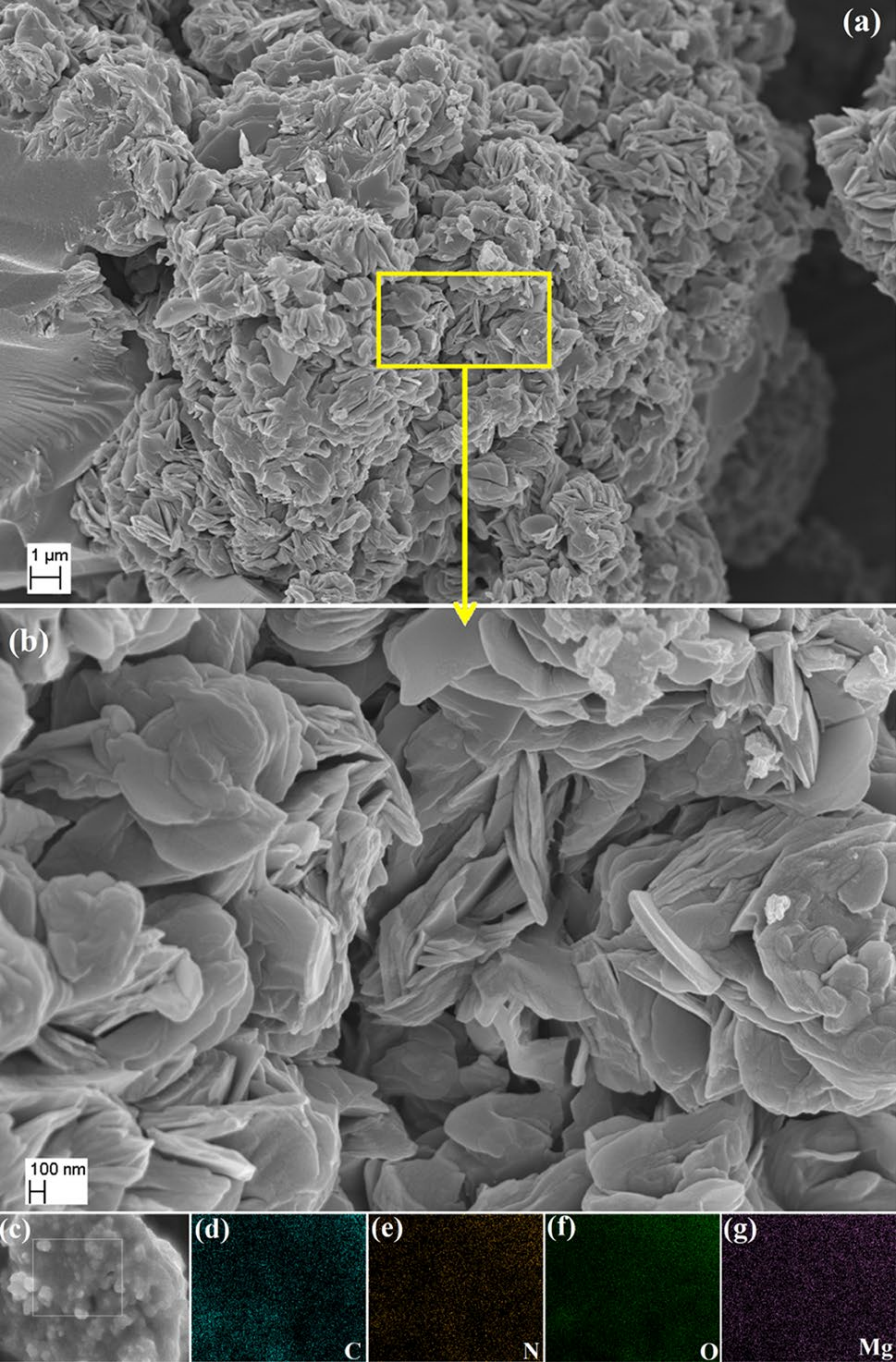
(**a**,**b**) The FESEM micrograph of Mg@TMA metallohydrogel, (**c**–**g**). EDX elemental mapping confirms the presence of primary chemical compositions i.e. C, N, O, and Cu elements.

The morphology pattern of TMA based Mg@TMA metallohydrogel was visualised by FESEM analysis. The FESEM pattern showed beautiful nano-rose type architecture of the metallohydrogel which are the output of self-assemble of metallohydrogel forming chemical components in presence of water solvent (Fig. 5a,b). The EDX elemental mapping analysis validates the existance of major elements C, N, O and Mg of Mg(NO_3_)_2_·6H_2_O, trimethylamine and water molecules, which forms the Mg@TMA metallohydrogel networks (Fig. 5c–g).

### FT-IR and powder X-ray diffraction study of Mg@TMA metallohydrogel

**Figure 6:**
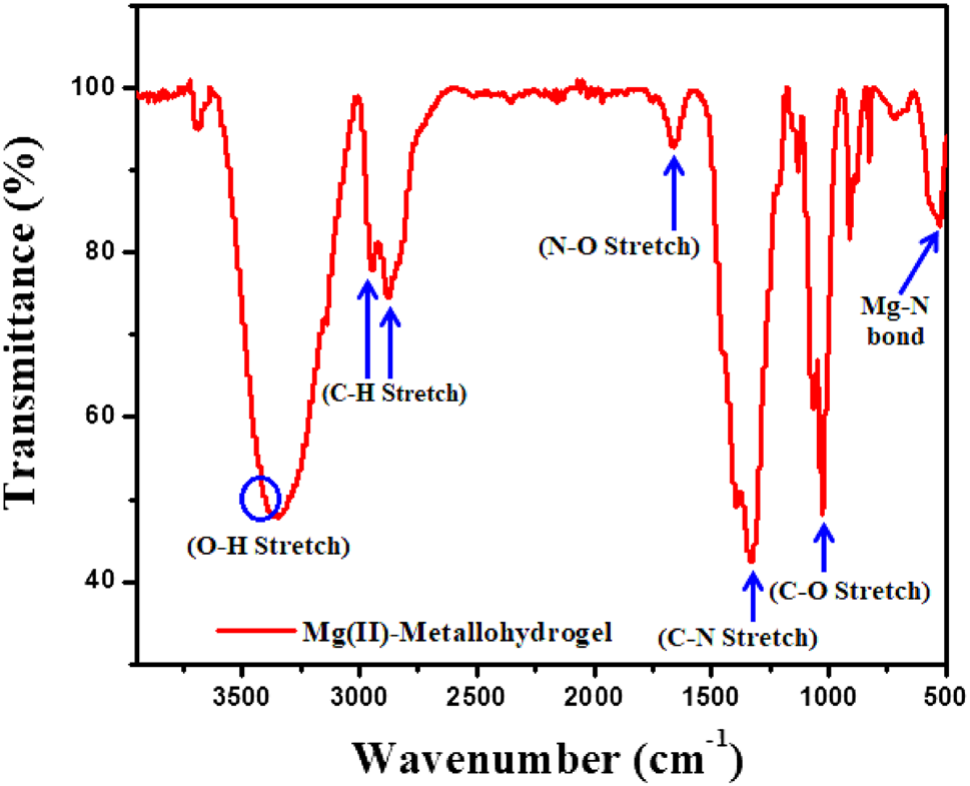
FT-IR spectra of the xerogel form of Mg@TMA metallohydrogel.

To characterise the synthesised Mg@TMA metallogel, Fourier Transform Infrared (FT-IR) spectroscopy was carried out because of its sensitivity towards different functional groups. Supramolecular interactions between trimethylamine and Mg(II)-source, which create the metallogel, are shown by the FT-IR spectra of Mg@TMA metallohydrogel in its xerogel form (Fig. 6). Significant absorption peaks are seen in the spectrum at ~ 3410 cm^−1^, 3340 cm^−1^, 2940 cm^−1^, 2870 cm^−1^, 1650 cm^−1^, 1330 cm^−1^, 1030 cm^−1^, 912 cm^−1^ and 527 cm^−1^. The O–H stretching vibrations are causing the significant broad peaks seen at 3410 cm^−1^ and 3340 cm^−1^. Both symmetric and asymmetric C–H bonds are responsible for the vibrational modes seen at 2870 cm^−1^ and 2940 cm^−1^. The peaks centred at 1650 cm^−1^, 1330 cm^−1^, 1030 cm^−1^ correspond to the N–O stretching, C–N stretching, C–O stretching vibrational modes, respectively. In addition, a peak at 527 cm^−1^ can be seen in the spectrum (Fig. 6), which is generated due to the existence of the Mg-N bond and supports the formation of the metallogel and demonstrates the strong interaction of trimethylamine with the water-soluble magnesium nitrate.

**Figure 7:**
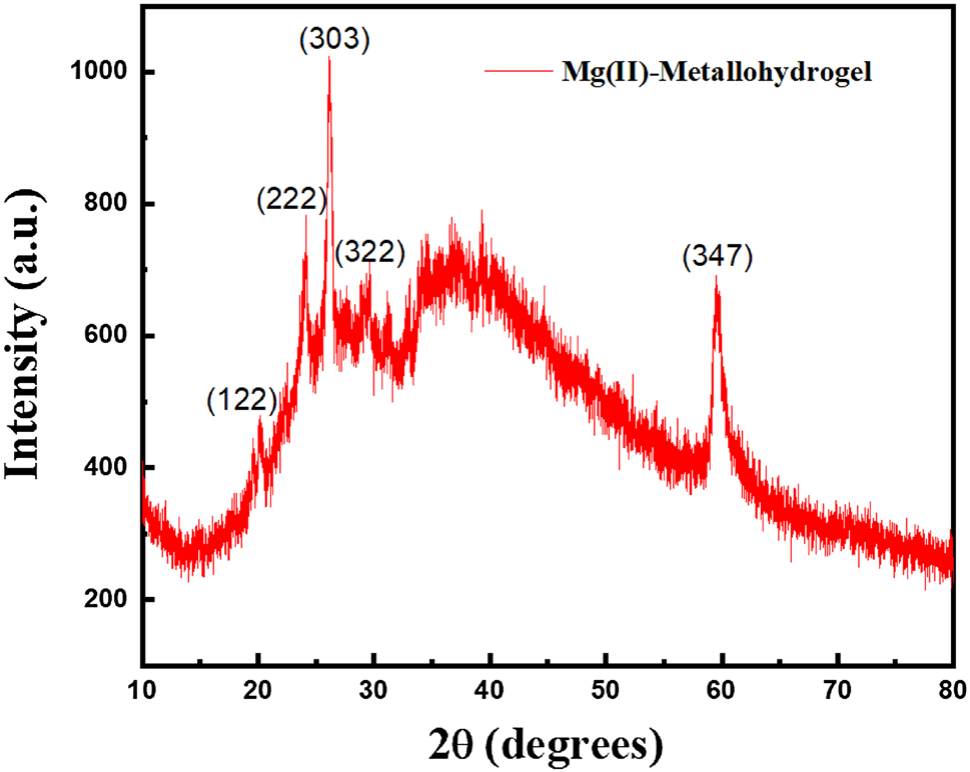
Powder X-Ray diffraction (PXRD) pattern of Mg@TMA in xerogel form.

The xerogel form of the Mg@TMA metallohydrogel is further analysed by powder X-ray powder diffraction method which indicates the nature of the sample. In Fig. 7, the PXRD data from angles 10–80° is shown. The presence of sharp peaks in the PXRD plot denotes that the synthesised metallogel has an amorphous structure. As a result, the application of electronic devices will be useful in other ways from the amorphous structure of Mg@TMA based metallogel.

### Optical characterization

**Figure 8:**
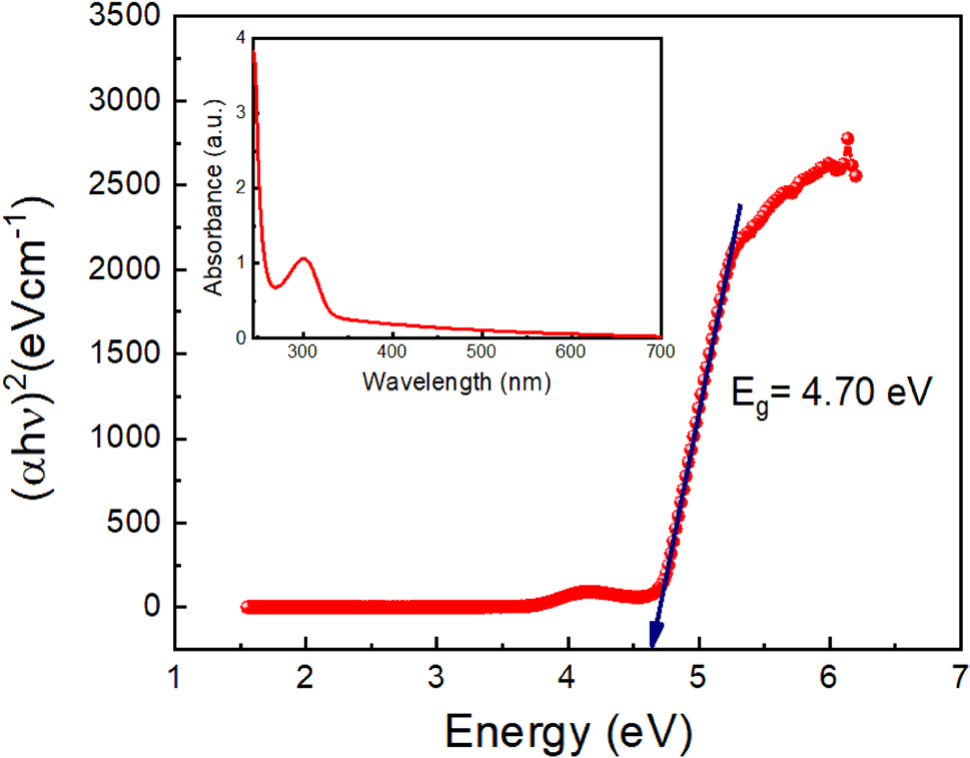
UV–Vis absorption spectra (inset) and Tuac's plots for Mg@TMA metallohydrogel.

The optical property of the synthesized metallogel Mg@TMA was investigated using UV–Vis absorption spectra, as shown in Fig. 8 (inset). The optical measurement was conducted in the wavelength range of 250–700 nm (refer to the UV–Vis absorption spectra). The optical bandgap (E_g_) of the Mg@TMA metallogel was calculated from the UV–Vis spectrum using Tauc's equation, which is given by:

1 $$\left( {\alpha h\nu } \right)^{n} = \, A \, \left( {h\nu - E_{g} } \right)$$

where α, E_g_, h, and ν are absorption coefficient, band gap, Planck’s constant, and frequency of light respectively. The exponent “n” is the electron transition processes dependent constant. “A” is a constant which is considered as 1 for ideal case. To determine the direct optical band gap, the value of the exponent “n” in the above equation was considered as n = 2. By extrapolating the linear region of the plot (αhν)^2^ vs hν (Fig. 8) to α = 0 absorption, we calculated the direct optical band gap (E_g_) which is 4.70 eV.

### Device fabrication

**Figure 9:**
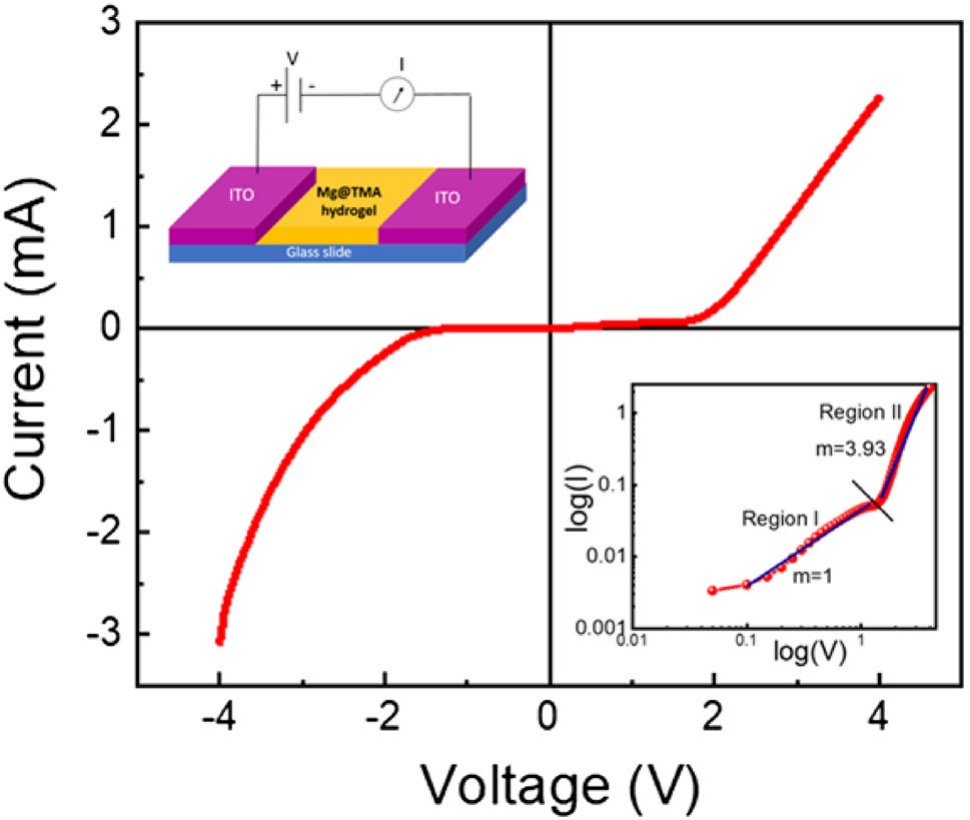
Schematic diagram of Mg@TMA metallohydrogel-based device (ITO/Mg@TMA/ITO) (left: inset) and I–V curve for (ITO/Mg@TMA/ITO) device at room temperature in linear scale and I–V curve in logarithmic scale (right : inset).

To perform the electrical characterization of the synthesized material, we fabricated a metal–semiconductor junction-based lateral Schottky diode-like structure in an ITO (Indium Tin Oxide)/Mg@TMA/ITO configuration (named as **device 1**), as shown in Fig. 9 (inset). To fabricate the junction device, we grew a thin film of as-synthesized Mg@TMA on a pre-cleaned glass substrate coated with ITO using the Doctor's blade method and subsequent annealing to remove the solvent part. ITO is highly conducting and optically transparent in the visible wavelengths, making it useful for studies involving photo-excitations.

In the second part of our work, RRAM (Resistive Random Access Memory) devices based on Mg@TMA metallohydrogel were fabricated in vertically sandwiched structures of Cu/Mg@TMA/Cu (named as **device 2**) and ITO/Mg@TMA/Cu (named as **device 3**) configurations to investigate the resistive switching behavior. In both configurations, the bottom electrodes were cleaned and Mg@TMA was deposited, after which the top electrode was placed.

### Electrical characterization

The presence of a band gap in Mg@TMA metallohydrogel confirms its semiconductor nature in thin film geometry, inspiring us to explore its charge transport properties for further device design. Figure 9 shows the I–V curve of device 1 on a linear scale, where no significant conduction is observed in the voltage range of (− 4 V to + 4 V). Beyond this range, the current rapidly increases with an increase in the applied voltage in both positive and negative polarity. The non-linear nature of the Schottky diode’s I–V curve was further analyzed using Cheung’s suggested method^101^ to extract key diode parameters, applying thermionic emission theory (TE Theory) to analyze the data. The observed I–V curve has been quantitatively analyzed by taking into account the subsequent equations.

2 $$I \, = I_{0} exp\left( {\frac{qV}{{\eta K_{B} T}}} \right)\left[ {1 - exp\left( {\frac{ - qV}{{\eta K_{B} T}}} \right)} \right]$$

3 $$I_{0} = A A^{* } T^{2 } \exp \left( {\frac{{ - q\phi_{B} }}{{K_{B} T}}} \right)$$

Here, I_0_ = Saturation Current, q = Electronic Charge, K_B_ = Boltzmann Constant, T = Temperature, V = Applied voltage, A = Effective diode area, η = Ideality factor, Φ_B_ = Barrier potential height, R_S_ = Series resistance, A* = Effective Richardson constant which is considered as 32 AK^−2^ cm^−2^ for this device.

To gain a detailed understanding of the conduction mechanism, we plotted the log I versus log V graph, which is shown in Fig. 9 (right: inset). This I–V graph on a log scale is divided into two voltage regions with different slopes. In region 1, the slope is equal to 1, and the current follows ohmic conduction in the lower voltage region. In region 2, when the voltage is increased, the slope is 3.93, indicating that the device follows the space charge limited conduction mechanism. We can conclude that when the slope is more than 2, it will follow the trap-filled Space Charge Limited Conduction (SCLC) process.

4 $$\frac{dV}{d({\text{ln}}I)}= \left(\frac{\eta {K}_{B}T}{q}\right)+I{R}_{S}$$

5 $$H\left(I\right)=V- \left(\frac{\eta {K}_{B}T}{q}\right){\text{ln}}\left(\frac{I}{A{A}^{*}{T}^{2}}\right)$$

6 $$H\left(I\right)=I{R}_{S}+ \eta {\phi }_{B}$$

**Figure 10:**
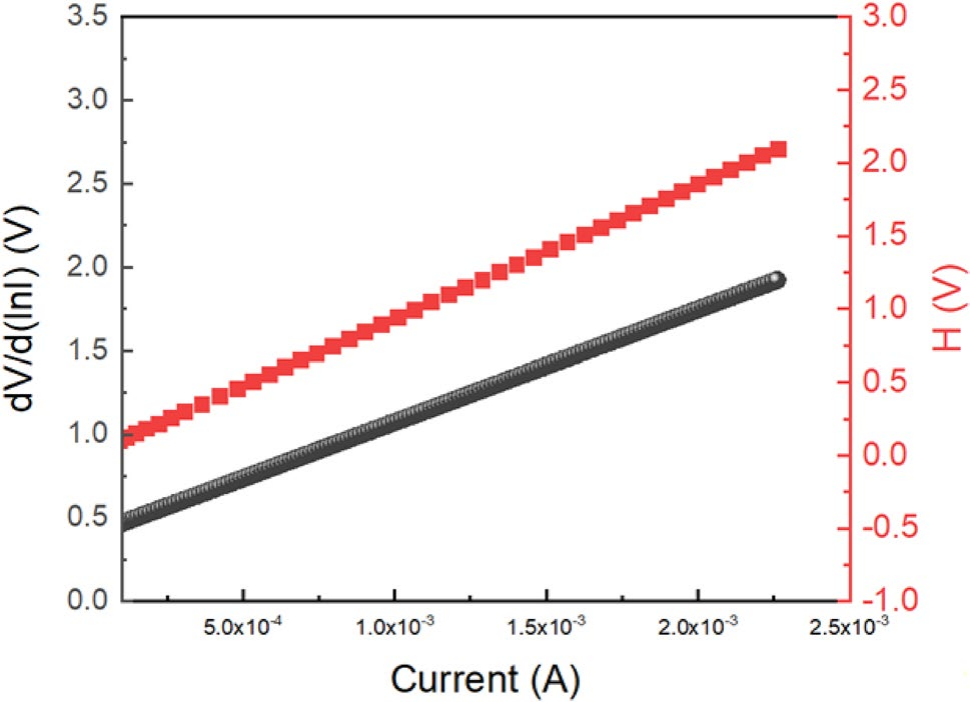
dV/d(ln I) versus I graph and the H versus I graph for ITO/Mg@TMA/ITO device.

Using Eqs. (4) through (6), which are taken directly from Cheung’s theory, we estimated the series resistance, ideality factor, and barrier potential height. To measure the diode parameters of the device 1, we plotted the dV/d(ln I) versus I graph and H versus I graph, as shown in Fig. 10. From the intercept of the dV/d(ln I) versus I graph, we calculated the ideality factor (η) and from the intercept of the H versus I graph, we determined the barrier height. The calculated ideality factor (η) for our diodes is 0.839, which is not ideal. The deviation from optimal behaviour may be caused by series resistance at the interface, the presence of interface states, and the existence of inhomogeneities in the Schottky barrier.

For device 1, the obtained value of the barrier height (Φ_B_) is 0.0258 eV, which is very low. Thus, we can conclude that the fabricated diode has a superior ideality factor and a lower barrier potential height, both of which are responsible for a Schottky diode. Additionally, we determined the value of the series resistance, which is 599.1575 Ω, from the slope of the dV/d(ln I) versus I graph and H versus I graph. The R_S_ value is similarly low, indicating that less power is being wasted. Such diode structures can be useful in the field of semiconductor devices, considering all of the measured properties.

**Figure 11:**
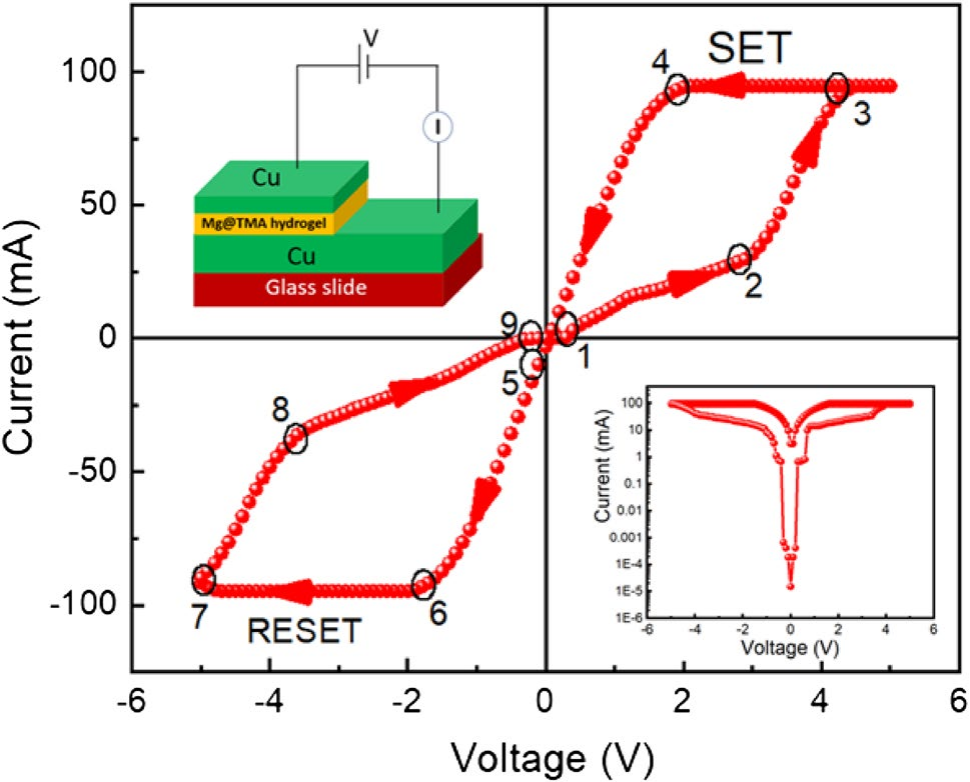
Schematic diagram of glass/Cu/Mg@TMA/Cu based device (left: inset) and I–V Curve of glass/Cu/Mg@TMA/Cu based device at linear scale (Here, point 1 → 2 denotes linear increase in current, 2 → 3 denotes rapid increase in current, 3 → 4 denotes current flows in reverse direction continuously, 4 → 5 denotes suddenly current decreases at 0 V, 5 → 6 denotes current decreases continuously upto − 1.78 V, 6 → 7 denotes current changes direction upto − 5 V, 7 → 8 denotes current increases rapidly upto − 3.64 V, 8 → 9 denotes current increases continuously upto − 0.1 V) and I–V Curve of glass/Cu/Mg@TMA/Cu based device at semi logarithmic scale (right: inset).

Next, the Resistive switching behaviour of Mg@TMA metallohydrogel based heterostructure is studied from IV measurement of device 2, where Cu acts as both bottom and top electrode (shown in Fig. 11 (inset)). Before starting measurements, compliance current (CC) is set at 100 mA to avoid any leakage contribution in IV measurement. Figure 11 shows the I–V curve of device 2 on a linear scale. Here the IV measurements are performed in the following sequence: 0 V → 5 V → to 0 V → − 5 V → 0 V. The arrows represent the direction of applied voltage. A complete hysteresis in the IV loop is observed, which is the signature of a memristor. Initially, close to 0 V, the current is very low, which increases almost linearly in the low-voltage region (point 1 to point 2). However, non-linearity in current–voltage characteristics is observed beyond a certain voltage (2.82 V), followed by the sudden jump of current on further increase in the applied voltage. It is called as the SET voltage (V_SET_), which in this case is 2.82 V that indicates the system goes from higher resistance state (HRS) to a lower resistance state (LRS) at this point. At point 3, the current becomes significantly large and the device goes to a complete ON state which remains the same on reversing the voltage from point 3 to point 4. On further reduction of the voltage, the current starts decreasing rapidly and goes to a high resistance state close to point 5, where the system goes to an OFF state. Upon continuing the cycling in the negative polarity, the current starts increasing and reaches an LRS at value = − 1.78 V and the sample goes back to its HRS at RESET voltage (V_*reset*_) ∼− 4.96 V. The process through which the system comes back to its HRS from LRS is called as the RESET process and is shown by the arrow between point 7 and point 8. This is a signature of bipolar resistive switching behaviour of the sample as a negative voltage is required to get the sample back into its previous resistance state. Further sweep in the voltage path results in a linear decrease in current from point 8 to point 9. Figure 11 (right: inset) shows the I–V characteristics of device 2 on a semi-logarithmic scale. This sudden increase and decrease in current are responsible for the formation of conductive filament-type structures which tends to switch the device between the ON and OFF states, respectively, which has been explained later. We have also measured the I–V curves of ITO/Mg@TMA/Cu based device and Cu/Mg@TMA/Cu based device upto 500 consecuitive cycles as shown in Figure S1 and Figure S2 respectively.

**Figure 12:**
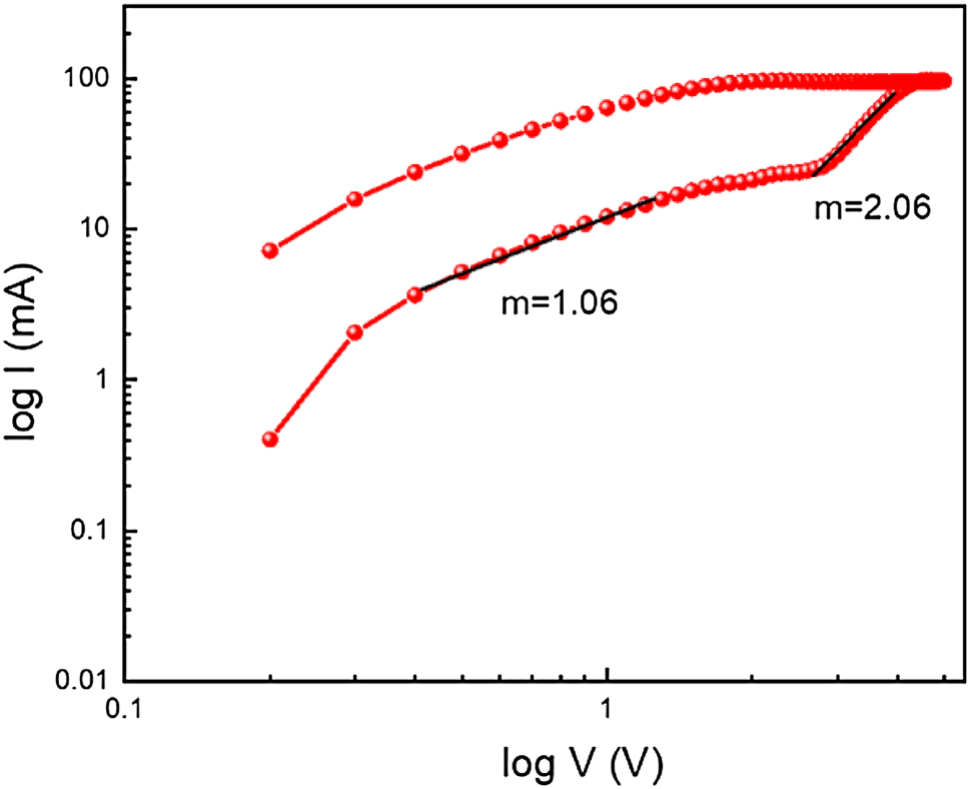
I–V Curve of glass/Cu/Mg@TMA/Cu based device at logarithmic scale.

To understand the charge transport mechanism of device 2, we have fitted the I–V curve in logarithmic scale (Fig. 12) during the SET process. Here, we observed that, the current varies linearly with a slope of *m* ~ 1.06 in voltage region between 0 and 2 V and this follows the Ohmic nature of conduction. But in the higher voltage region between 2 and 3 V, the slope, *m* ~ 2.06, which could arise due to Cu^2+^ ion migration in higher voltage region. The complete range of I–V curve indicates that the device is followed space charge conduction mechanism.

**Figure 13:**
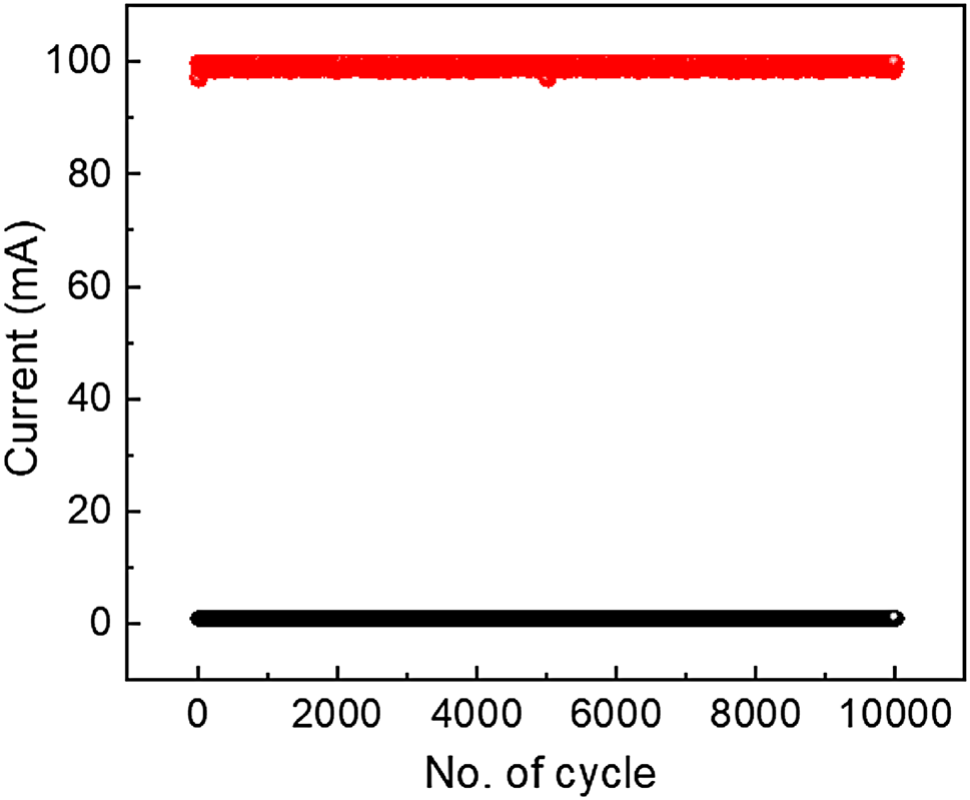
Endurance test of glass/Cu/Mg@TMA/Cu based device.

To understand the robustness of the switching process, we have performed the switching measurements repeatedly for 10,000 cycles of operation at room temperature as shown in Fig. 13. From the endurance test, it is observed that switching operation in device 2 is stable upto 10,000 switching cycles with an average ON/OFF ratio ~ 100. It suggests that this device retains its memory for a long time without any degradation which can be useful for practical applications in swift memory circuit design at a reduced cost of fabrication.

**Figure 14:**
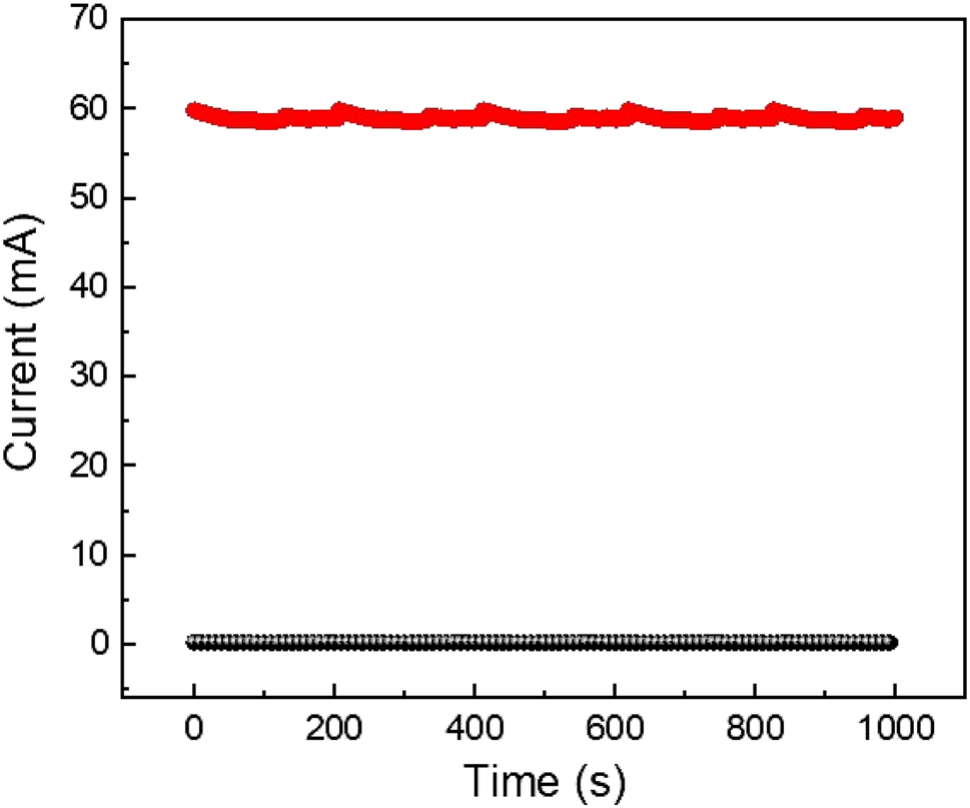
Retention test of glass/Cu/Mg@TMA/Cu based device.

We have also performed retention test upto 10^3^ s for the device 2 at room temperature as shown in Fig. 14. The switching procedure for device 2 is stable up to 10^3^ s. Here, we have observed that for this device ON/OFF ratio is around 60. So, we can conclude that this device can retain data upto 10^3^ s without any degradation.

**Figure 15:**
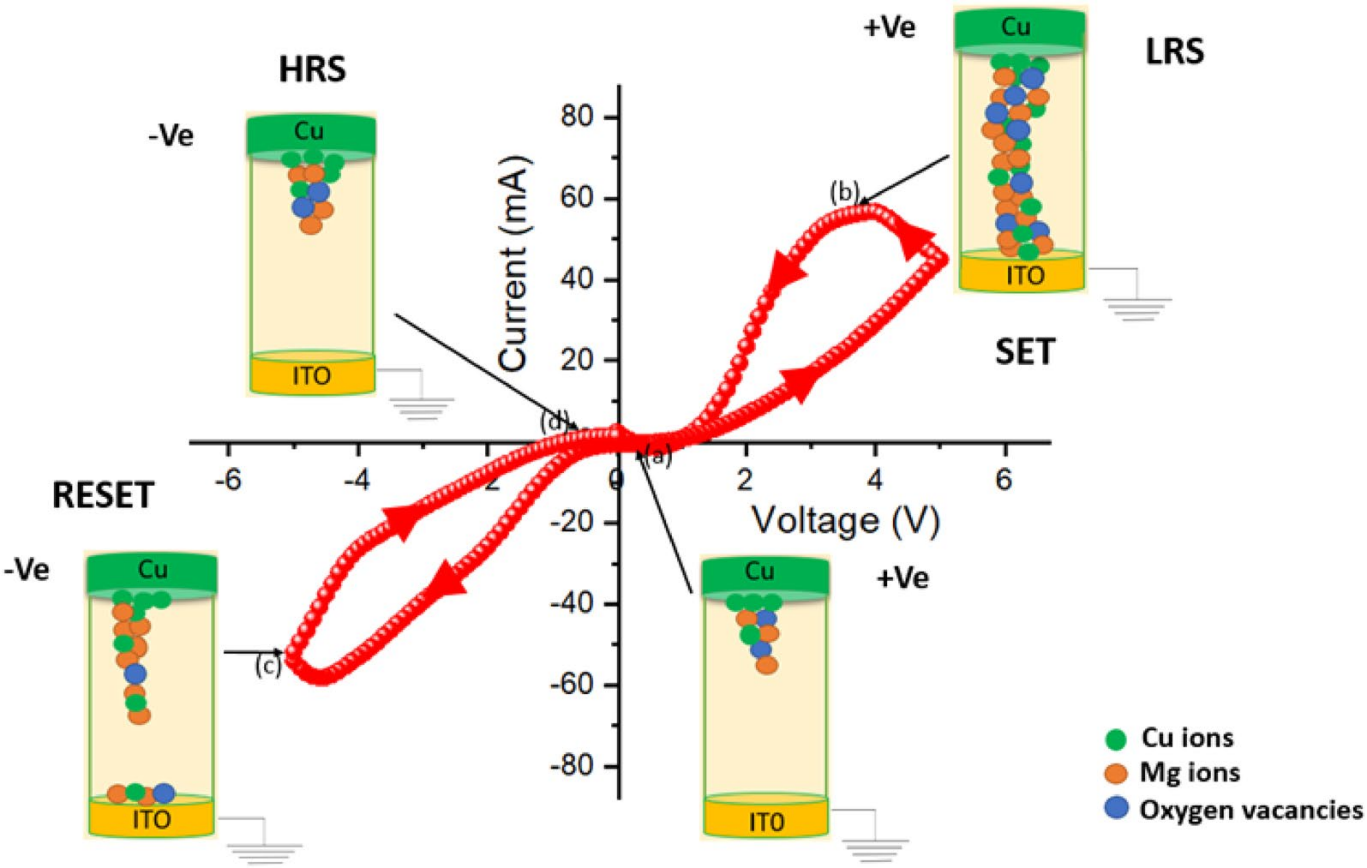
A schematic diagram of the conductive filament model demonstrating the resistive switching mechanism of glass/ITO/Mg@TMA/Cu based device. Various points along the I–V curve has been marked as follows: (**a**) The migration of the Cu ions, Mg ions and oxygen vacancies towards the intermediate layer after the application of the positive voltage of 0.2 V, (**b**) LRS state in which conducting filament type structure formed with Cu ion, Mg ions, oxygen vacancies at 3.8 V, (**c**) Rupture of the conducting filament in RESET process and Cu ions, Mg ions, oxygen vacancies are going back to the top electrode by the application of negative voltage of − 5 V, (**d**) All ions are accumulated in the top electrode and switch to HRS at − 0.2 V. A comparison of I_ON_/I_OFF_ observed in other material at room temperature (shown in Table 1).

We have also investigated the resistive switching behaviour of device 3 (ITO/Mg@TMA/Cu), where ITO acts as a bottom electrode and Cu acts as a top electrode. The I–V curve shows memristive behaviour over complete voltage cycling, with the presence of distinct LRS and HRS states through the SET and RESET processes (V_SET_ = 5.1 V and V_RESET_ = − 5.2 V) as shown in Fig. 15, similar to the observation made for device 2.

To better understand the consistency of the switching process, we also performed an endurance test over 5000 switching cycles consecutively at room temperature for the device 3 (ITO/Mg@TMA/Cu), as shown in Figure S5. The switching procedure for this device is reliable for up to 5000 switching cycles respectively. The endurance test shows that the switching behaviour is robust because the average ON/OFF ratio is around 60 for this device. It implies that this device can continue to function as intended in terms of memory response over an extended period without experiencing any degradation.

We have also performed retention test upto 10^3^ s for the device 3 (ITO/Mg@TMA/Cu) at room temperature as shown in Figure S6. The switching procedure for this device is stable up to 10^3^ s. Here, we have observed that for this device ON/OFF ratio is around 100. So, we can conclude that this device can retain data upto 10^3^ s without any degradation.

The physical origin behind the switching process can be explained with the help of different processes: formation of Schottky barrier with electrochemical migration, redox reactions, valance change memory etc. In our work, electrochemical metallization mechanism (ECM) and valance change memory (VCM) depend on the motion of metal cations and oxygen defects. Here, it is observed that metal ions and oxygen vacancies play an important role for changing the state of resistance (Fig. 15). For device 3, the migration of Cu ions, Mg ions and oxygen vacancies results in metallic filament development in the semiconducting layers. By breaking and reforming the imbedded Cu filaments in the semiconducting layers, we are able to explain the change of resistance from HRS to LRS. In this study, the resistive switching behaviour of device 3 is caused by Cu ions and oxygen vacancies mainly. We already know that under an electric field, Cu ions can ionize to form Cu ions with the formula, Cu → Cu^2+^  + e−, and that Cu ions can travel in the direction of the applied electric field. When we apply positive voltage, Cu^2+^ ions, Mg ions and oxygen vacancies move towards the intermediate layer, where they are reduced to metallic Cu. Then, the conductivity of this layer will increase and the Cu ions, Mg ions and oxygen vacancies reach a particular level of accumulation towards the bottom electrode because they will act as conductive filaments to complete the SET process and switch from HRS to LRS. The device remains in the LRS state unless a sufficient voltage with opposite parity is applied to electrochemically dissolve the Cu filaments and oxygen vacancies for the RESET process. When negative voltage is applied, the device enters the HRS state and the conductivity of this device decreases simultaneously. Finally, Cu^2+^ ions, Mg ions and oxygen vacancies drift back to the top electrode. In a similar way, the resistive switching behaviour of the device 2 can be explained through the migration of Cu^2+^ ions, Mg ions and oxygen vacancies. We have also discussed the Cu ion migration using TEM and EDAX analysis shown in Figure S3 and S4.

**Figure 16:**
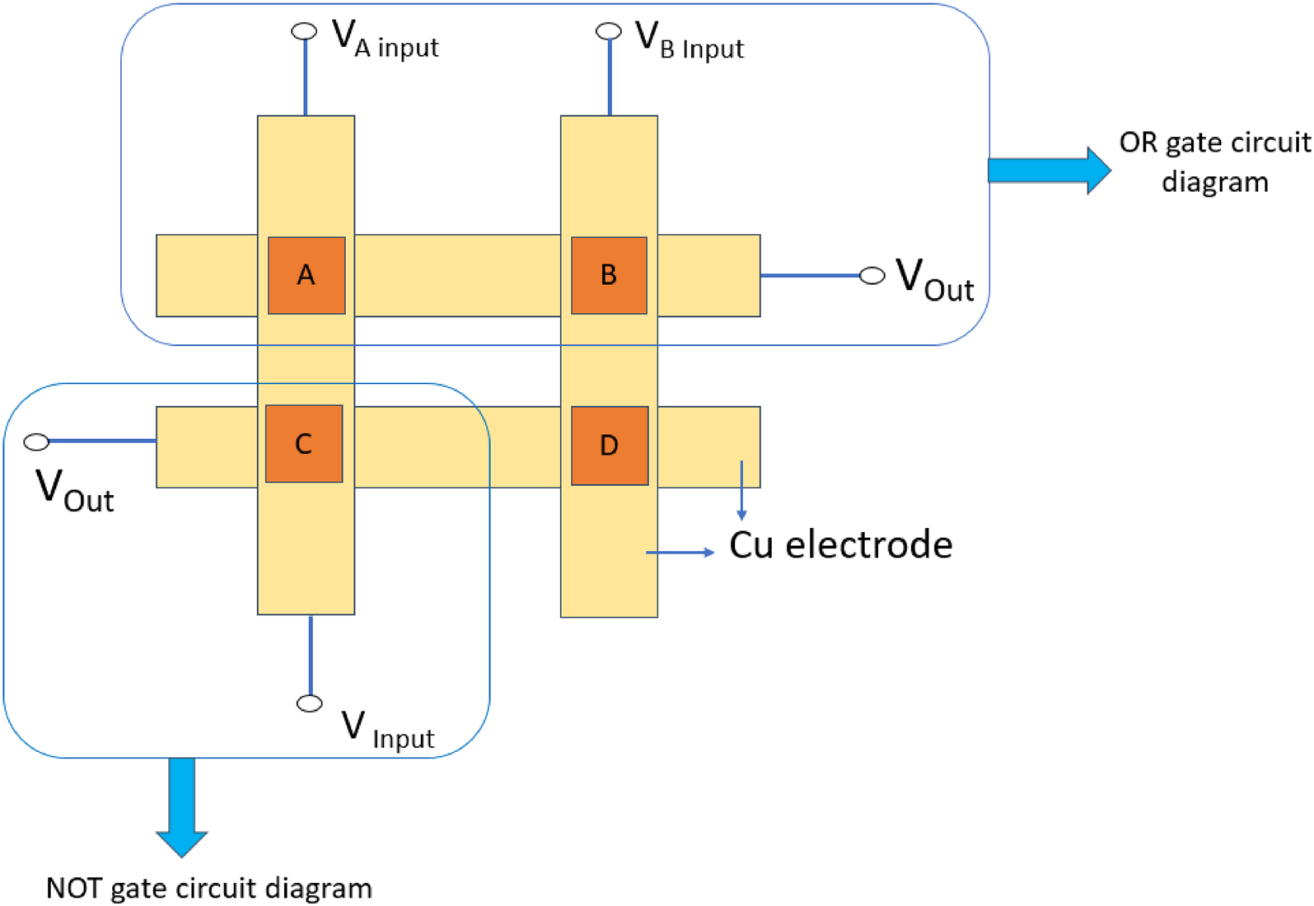
Schematic circuit diagram for OR gate and NOT gate.

Memory devices in memory systems are organized in arrays, and one common architecture is the cross-point architecture. In this configuration, our sample is placed at the crossing point of two perpendicular metal lines, as shown in Fig. 16. In our research work, we have shown how metallogel based RRAM device in crossbar array works in memory computing using logic gate operation. In-memory computing can also be accomplished by employing fundamental electrical circuit principles, such as Kirchhoff's law and Ohm's law. This method is particularly well-suited for analog crossbar arrays. Here, in this work we have prepared 2 × 2 cross bar device based on Cu/Mg@TMA/Cu structure where Cu acts as both top and bottom electrode as shown in Fig. 16 and four RRAM devices are denoted by A,B,C, D.

Now, for logic gate operations we have used two RRAM devices (A and B). For OR logic gate, if memristors A and B are both logical “0” that means no voltage is applied, then output voltage is 0.04 V which is considered as “0” state (Table 1). When we applied 5 V at Input A and 0 V at Input B, then output voltage is 4.86 V which is considered as logical “1” state. Similarly. when we applied 0 V at Input A and 5 V at Input B, then output voltage is 4.85 V which is also considered as logical “1” state. When both memristors A and B are both logical “1” state, that means when we applied 5 V at both the terminals, then the output voltage is also 4.85 V as logical “1” state. The truth table of OR logic gate is shown in the following Table 2.

**Table 1:** Comparison of SET and RESET values with different materials.

System	V_SET_ (V)	V _RESET_ (V)	I_ON_ / I_OFF_	Reference
ITO/(PF_6_)_4_/Al	3.4	− 4.3	100	^98^
Al/PMMA/Al	1.4 to 3.9	− 0.15 to − 1.7	10^7^	^99^
Al/Ag/PMMA/Al	1.8 to 3.6	− 0.28 to − 1.8	10^5^	^99^
Al/Ag/AgNP-PMMA/Al	1.1 to 3.2	− 0.09 to − 0.6	10^5^	^99^
Ag/CD-MOF/Ag	± 7.5	± 1.5	30	^100^
Ag/FJU-23-H2O/Ag	0.2	–	10^5^	^100^
ITO/NPs@ MOF film/Ag	2.1	− 2.1	10^4^	^100^
Ag/ZIF-8/Si	1.80 to 2.65	− 0.80 to − 2.35	10^4^	^100^

**Table 2:** Truth table of OR gate.

Voltage at input A (V)	Voltage at input B (V)	Output voltage (V)	Logical state
0	0	0.04	0
5	0	4.86	1
0	5	4.85	1
5	5	4.85	1

Similarly, we have designed NOT gate logic circuit (Fig. 16) using device C and it is also satisfied NOT gate truth table which is shown in the following Table 3.

**Table 3:** Truth table of NOT gate.

Input voltage (V)	Output voltage (V)	Logical state
0	4.81	1
5	0.03	0

The current structure can be extended further with a larger size of cross-point arrays to perform advanced logic and computing operations, which can act as a central part for in-memory computing where the computation and information storage are done at the same circuit level, as demonstrated here. In this way, memristor based logic gate circuits using crossbar arrays will help us to explore different engineering methodologies that depends on in-memory computing principles.

## Conclusions

In summary, an aliphatic amine trimethylamine gelator based supramolecular Mg(II)-metallohydrogel has been produced through the rapid mixing of water solution of magnesium nitrate and trimethylamine at room temperature. The Mg(II)-metallohydrogel is stable for months at room temperature because of a variety of non-covalent interactions. By using FESEM, the unique nano-rose like shape of Mg(II)-metallohydrogel was investigated. Rheological experiment has confirmed the Mg(II)-metallohydrogel’s mechanical stability. Both Fourier transform infrared and powder X-ray diffraction spectroscopy are used to analyse potential intermolecular interactions. Our synthesised Mg(II)-metallohydrogel’s optical band-gap measurement is indicative of its semiconducting nature. We have also manufactured a metal–semiconductor junction based device with lateral and vertical structures made from Cu, ITO, and the semiconducting Mg(II)-metallohydrogel. The I–V characteristic acquired from this method of manufacturing verifies that the device is really a Schottky diode and provides evidence of the nonlinear charge transport it employs. In this work, we have also fabricated RRAM devices in ITO/Mg@TMA/Cu and Cu/Mg@TMA/Cu heterostructures. For both the devices, we have observed robust bipolar resistive switching behaviour originating from the formation and rupture of conduction filaments between the two electrodes. The switching process results in the appearance of distinct ON and OFF states which remains stable over 10,000 switching cycles with an average ON/OFF ratio ~ 100, demonstrating the device's non-volatile nature and superior reliability. It confirms that the Mg(II)-metallohydrogel is a preferable candidate for non-volatile memory application due to its stable switching behaviour, robust endurance, offering multi-functionality of applications in neuromorphic computing and switching neural circuit design. In addition, it inspires us to design flexible electronic device from Mg@TMA metallohydrogel-based thin films in printable electronics for large scale assembly in advanced technological applications.

## Experimental section

### Materials

Magnesium(II) nitrate hexahydrate and trimethylamine were purchased from Tokyo Chemical Industry (TCI) and used without any further purification. Double-distilled water was used throughout the work.

### Characterizations

SHIMADZU UV-3101PC UV–visible spectrometer is used to acquire the absorption data.

An Anton Paar 100 rheometer with a cone and plate geometry (CP 25-2) and an adjustable peltier temperature regulating device was used to carry out all of the rheological tests.

The ZEISS Gemini SEM 400 field emission scanning electron microscope (FESEM) was used to study the microstructure of the synthesized gel material.

The metallogel’s FTIR spectrum was obtained using a JASCO FTIR 4700 spectrometer.

Powder X-ray diffraction (PXRD) study was accompanied using PANALYTICAL X'Pert Powder X-Ray diffractometer with CuK_α1_ radiation.

Keithley 2400 source meter connected via Labview was utilized to study the current–voltage (I–V) measurements of synthesized metallogel-based devices in a two-probe configuration at room temperature.

### Supplementary Information

Supplementary Information.

## Data Availability

The datasets used and/or analysed during the current study available from the corresponding author on reasonable request.

## References

[1] Sangeetha NM (2005). Supramolecular gels: Functions and uses. Chem. Soc. Rev..

[2] Dastidar P (2008). Supramolecular gelling agents: Can they be designed?. Chem. Soc. Rev..

[3] Dhibar S (2019). Development of supramolecular semiconducting Mn(II)-metallogel based active device with substantial carrier diffusion length. ACS Appl. Electron. Mater..

[4] Karmakar K (2023). Instantaneous gelation of a self-healable wide-bandgap semiconducting supramolecular Mg(II)-metallohydrogel: An efficient nonvolatile memory design with supreme endurance. ACS Appl. Electron. Mater..

[5] Feldner T (2016). Supramolecular metallogel that imparts self-healing properties to other gel networks. Chem. Mater..

[6] Mitsumoto K (2017). A multi-redox responsive cyanometalate-based metallogel. Chem. Eur. J..

[7] Dhibar S (2019). A semiconducting supramolecular Co(II)-metallohydrogel: An efficient catalyst for single-pot aryl–S bond formation at room temperature. Dalton Trans..

[8] Dhibar S (2020). Triethylenetetramine-based semiconducting Fe(III) metallogel: Effective catalyst for Aryl–S coupling. ACS Omega.

[9] Xing B (2002). Design of coordination polymer gels as stable catalytic systems. Chem.-Eur. J..

[10] Huang J (2010). Dynamic functionalised metallogel: An approach to immobilised catalysis with improved activity. J. Mol. Catal. A.

[11] Sarkar S (2014). Redox-switchable copper(I) metallogel: A metal-organic material for selective and naked-eye sensing of picric acid. ACS Appl. Mater. Interfaces.

[12] Lin Q (2015). A novel supramolecular metallogel-based high-resolution anion sensor array. Chem. Commun..

[13] Gou F (2016). Unusual aggregation/gelation-induced phosphorescence of propeller-type binuclear platinum(II) enantiomers. Eur. J. Inorg. Chem..

[14] Kelly N (2016). Self-assembly of [2+2] Co(II) metallomacrocycles and Ni(II) metallogels with novel bis(pyridylimine) ligands. J. Organomet. Chem..

[15] Weiss RG (2005). Molecular Gels: Materials with Self-Assembled Fibrillar Networks.

[16] Tam AY-Y (2013). Recent advances in metallogels. Chem. Soc. Rev..

[17] Tomasini C (2013). Peptides and peptidomimetics that behave as low molecular weight gelators. Chem. Soc. Rev..

[18] Meazza L (2013). Halogen-bonding-triggered supramolecular gel formation. Nat. Chem..

[19] Wang Q (2010). Nature.

[20] Holten-Andersen N (2011). pH-induced metal-ligand cross-links inspired by mussel yield self-healing polymer networks with near-covalent elastic moduli. Proc. Natl. Acad. Sci..

[21] Burnworth M (2011). Optically healable supramolecular polymers. Nature.

[22] Cordier P (2008). Self-healing and thermoreversible rubber from supramolecular assembly. Nature.

[23] Shirakawa M (2003). Fullerene-motivated organogel formation in a porphyrin derivative bearing programmed hydrogen-bonding sites. J. Am. Chem. Soc..

[24] Park T (2006). Formation of a miscible supramolecular polymer blend through self-assembly mediated by a quadruply hydrogen-bonded heterocomplex. J. Am. Chem. Soc..

[25] Moffat JR, Seeley GJ, Carter JT, Burgess A, Smith DK (2008). Nanostructured polymers with embedded self-assembled reactive gel networks. Chem. Commun..

[26] Fages, F. *et al.* 77–131 (Springer, 2005).

[27] Al-Dossary M (2018). Copper-based hydrogels with dicarboxylate spacer ligands for selective carbon dioxide separation applications. New J. Chem..

[28] Jung JH (2001). Self-assembly of a sugar-based gelator in water: Its remarkable diversity in gelation ability and aggregate structure. Langmuir.

[29] Rajkamal R (2016). Arabinose based gelators: Rheological characterization of the gels and phase selective organogelation of crude-oil. RSC Adv..

[30] Chalard A (2018). Simple synthetic molecular hydrogels from self-assembling alkylgalactonamides as Scaffold for 3D neuronal cell growth. ACS Appl. Mater. Interfaces.

[31] Draper ER (2016). Photoresponsive gelators. Chem. Commun..

[32] Dhibar S (2019). The development of a rapid self-healing semiconducting monoethanolamine-based Mg(OH)_2_ metallogel for a Schottky diode application with a high ON/OFF ratio. New J. Chem..

[33] Alam N (2020). A thixotropic supramolecular metallogel with a 2D sheet morphology: Iodine sequestration and column based dye separation. Soft Matter.

[34] Svobodová H (2012). Recent advances in steroidal supramolecular gels. RSC Adv..

[35] Gao L (2014). Construction of supramolecular organogels and hydrogels from crown ether based unsymmetric bolaamphiphiles. Chem. Commun..

[36] Karan CK (2016). Self-healing and moldable metallogels as the recyclable materials for selective dye adsorption and separation. ACS Appl. Mater. Interfaces.

[37] Dey S (2013). A coordination-assisted general approach to nickel-based nano metallogels. RSC Adv..

[38] Piepenbrock M-OM (2010). Shear induced gelation in a copper(II) metallogel: New aspects of ion-tunable rheology and gel-reformation by external chemical stimuli. Soft Matter.

[39] Piepenbrock M-OM (2009). Metal ion and anion-based “tuning” of a supramolecular metallogel. Langmuir.

[40] Lin Q (2014). Competitive coordination control of the AIE and micro states of supramolecular gel: An efficient approach for reversible dual-channel stimuli-response materials. Soft Matter.

[41] Amacher AM (2015). Coordination-directed self-assembly of a simple benzothiadiazole-fused tetrathiafulvalene to low-bandgap metallogels. Chem. Commun..

[42] Sarkar S (2014). Redox-responsive copper(I) metallogel: A metal-organic hybrid sorbent for reductive removal of chromium(VI) from aqueous solution. Langmuir.

[43] Dhibar S (2018). A supramolecular Cd(II)-metallogel: An efficient semiconductive electronic device. Dalton Trans..

[44] Dhibar S (2019). A supramolecular gel of oxalic acid-monoethanolamine for potential Schottky barrier diode application. ChemistrySelect.

[45] Dhibar S (2020). Organic-acid-mediated luminescent supramolecular Tb(III)-metallogel applied in an efficient photosensitive electronic device with excellent charge transport properties. Ind. Eng. Chem. Res..

[46] Dhibar S (2020). Terephthalic acid-directed supramolecular Cu(II)-metallogel for photosensitive semiconducting Schottky diode with promising electronic charge transportation. Int. J. Energy Res..

[47] Ganta S (2015). Nanoscale metallogel via self-assembly of self-assembled trinuclear coordination rings: Multi-stimuli-responsive soft materials. Dalton Trans..

[48] Yang L (2010). Self-assembly from metal–organic vesicles to globular networks: Metallogel-mediated phenylation of indole with phenyl boronic acid. Chem. Commun..

[49] Xing B (2002). Spontaneous enrichment of organic molecules from aqueous and gas phases into a stable metallogel. Langmuir.

[50] Ma X (2017). A novel thermo-responsive supramolecular organogel based on dual acylhydrazone: Fluorescent detection for Al^3+^ ions. Soft Matter.

[51] Jiang B (2017). Multiphase transition of supramolecular metallogels triggered by temperature. Chem. Commun..

[52] Po C (2013). A Platinum(II) terpyridine metallogel with an L-valine-modified alkynyl ligand: Interplay of Pt⋅⋅⋅Pt, π–π and hydrogen-bonding interactions. Chem. Eur. J..

[53] Ganta S (2018). Multi-stimuli-responsive metallogel molded from a Pd2L4-type coordination cage: Selective removal of anionic dyes. Inorg. Chem..

[54] Chen P (2015). White-light-emitting lanthanide metallogels with tunable luminescence and reversible stimuli-responsive properties. J. Am. Chem. Soc..

[55] Kelly N (2016). Self-assembly of [2 + 2] Co(II) metallomacrocycles and Ni(II) metallogels with novel bis (pyridylimine) ligands. Organomet. Chem..

[56] Offiler CA (2017). Metal ‘turn-off’, anion ‘turn-on’ gelation cascade in pyridinylmethyl ureas. Chem. Commun..

[57] Dhibar S (2019). Development of supramolecular semiconducting Mn(II)-metallogel based active device with substantial carrier diffusion length. ACS Appl. Mater. Interfaces.

[58] Saha S (2013). Proton-conducting supramolecular metallogels from the lowest molecular weight assembler ligand: A quote for simplicity. Chem. Eur. J..

[59] Aiyappa HB (2015). Fe(III) phytate metallogel as a prototype anhydrous, intermediate temperature proton conductor. Chem. Sci..

[60] Smith LJ (2018). Diels-alder click-cross-linked hydrogels with increased reactivity enable 3D cell encapsulation. Biomacromolecules.

[61] Drury JL (2003). Hydrogels for tissue engineering: Scaffold design variables and applications. Biomaterials.

[62] Caló E (2015). Biomedical applications of hydrogels: A review of patents and commercial products. Eur. Polym. J..

[63] Dhibar S (2023). A transparent self-healable multistimuli-responsive novel supramolecular Co(II)-metallogel derived from adipic acid: Effective hole transport layer for polymer solar cells. J. Mol. Liq..

[64] Schroeder TBH (2017). An electric-eel-inspired soft power source from stacked hydrogels. Nature.

[65] Mukhopadhyay RD (2018). Stepwise control of host–guest interaction using a coordination polymer gel. Nat. Commun..

[66] Kurbah SD (2020). Vanadium(V) complex based supramolecular metallogel: Self-assembly and (Metallo)gelation triggered by non-covalent and N^+^single bond H…O hydrogen bonding interactions. Inorg. Chem. Commun..

[67] Chen J (2016). A hydro-metallogel of an amphiphilic L-histidine with ferric ions: Shear-triggered self-healing and shrinkage. Inorg. Chem. Front..

[68] Zhong J-L (2016). Self-assembled metallogels formed from N, N′, N′′-tris (4-pyridyl) trimesic amide in aqueous solution induced by Fe(III)/Fe(II) ions. Soft Matter.

[69] Arnedo-Sánchez L (2017). Rapid self-healing and anion selectivity in metallosupramolecular gels assisted by fluorine–fluorine interactions. Dalton Trans..

[70] Yan L (2018). Visual discrimination of 2-picolinic acid by a supramolecular metallogel. Chem. Lett..

[71] Lee JH (2016). Metallogel of bis(tetrazole)-appended pyridine derivative with CoBr_2_ as a chemoprobe for volatile gases containing chloride atom. Supramol. Chem..

[72] Wang X (2012). Universal chiral twist via metal ion induction in the organogel of terephthalic acid substituted amphiphilic L-glutamide. Chem. Commun..

[73] Das P (2021). 4, 4′-Bipyridine-based Ni(II)-metallogel for fabricating a photo-responsive Schottky barrier diode device. New J. Chem..

[74] Dhibar S (2023). An organic acid consisted multiresponsive self-healing supramolecular Cu(II)-metallogel: Fabrication and analysis of semiconducting device. J. Mol. Liq..

[75] Dhibar S (2022). A multistimulus-responsive self-healable supramolecular copper(II)-metallogel derived from l-(+) tartaric acid: An efficient Schottky barrier diode. New J. Chem..

[76] Dhibar S (2023). A novel citric acid facilitated supramolecular zinc(II)-metallogel: Toward semiconducting device applications. J. Mol. Liq..

[77] Karmakar K (2023). A novel supramolecular Zn(II)-metallogel: An efficient microelectronic semiconducting device application. RSC Adv..

[78] Majumdar S (2020). Cd-based metallohydrogel composites with graphene oxide, MoS_2_, MoSe_2_, and WS_2_ for semiconducting Schottky barrier diodes. ACS Appl. Nano Mater..

[79] Roy M (2022). Toxic metal–organic gels using a unique pyridine–pyrazole based ligand with Pb(II), Cd(II) and Hg(II) salts: Multi-stimuli responsiveness and toxic dye adsorption. Mater. Adv..

[80] Sebastian A (2019). A mixed ligand approach towards lanthanide-based gels using citric acid as assembler ligand: White light emission and environmental sensing. Soft Matter.

[81] Alam N (2023). Self-templated conversion of a self-healing metallogel into an active carbon quasiaerogel: Boosting photocatalytic CO_2_ reduction by water. ACS Sustain. Chem. Eng..

[82] Dastidar P (2016). Metallogels from coordination complexes, organometallic, and coordination polymers. Chem. Asian J..

[83] Alam N (2022). A wide bandgap semiconducting magnesium hydrogel: Moisture harvest, iodine sequestration, and resistive switching. Langmuir.

[84] Leong WL (2008). Fluorescent magnesium(II) coordination polymeric hydrogel. Chem. Eur. J..

[85] Xie J (2016). Luminescence properties of a thermal-responsive organogel to various metal cations. Inorg. Chem. Commun..

[86] Kumari K (2020). Structural and resistive switching behaviour in lanthanum strontium manganite - Reduced graphene oxide nanocomposite system. J. Alloys Compd..

[87] Kumari K (2021). Charge transport and resistive switching in a 2D hybrid interface. Mater. Res. Bull..

[88] Majumder S (2021). Pulsed voltage induced resistive switching behavior of copper iodide and La_0.7_Sr_0.3_MnO_3_ nanocomposites. Mater. Lett..

[89] Chang T-C (2016). Resistance random access memory. Mater. Today.

[90] Krishnan K (2021). Configurable switching behavior in polymer-based resistive memories by adopting unique electrode/electrolyte arrangement. RSC Adv..

[91] Zahoor F (2020). Resistive random access memory (RRAM): An overview of materials, switching mechanism, performance, multilevel cell (mlc) storage, modeling, and applications. Nanoscale Res. Lett..

[92] Kumari K (2021). Temperature-dependent resistive switching behaviour of an oxide memristor. Mater. Lett..

[93] Kumari K (2022). Resistive switching phenomena: A probe for the tracing of secondary phase in manganite. Appl. Phys. A.

[94] Kumari K (2021). The effect of graphene and reduced graphene oxide on the resistive switching behavior of La_0.7_Ba_0.3_MnO_3_. Mater. Today Commun..

[95] Kumari K (2022). Structural, resistive switching and charge transport behaviour of (1–x)La_0.7_Sr_0.3_MnO_3(x)_ZnO composite system. Appl. Phys. A.

[96] Kumari K (2022). Role of an oxide interface in a resistive switch. Curr. Appl. Phys..

[97] Majumder S (2023). Temperature-dependent resistive switching behavior of a hybrid semiconductor-oxide planar system. Appl. Phys. A.

[98] Kumar S (2022). Y_2_O_3_-based crossbar array for analog and neuromorphic computation. IEEE Trans. Electron Devices.

[99] Kumar S (2022). electrical performance of large-area Y_2_O_3_ memristive crossbar array with ultralow C2C variability. IEEE Trans. Electron Devices.

[100] Jadhav R (2020). Benzoselenadiazole-based conjugated molecules: Active switching layers with nanofibrous morphology for nonvolatile organic resistive memory devices. Chem. Plus Chem..

[101] Cheung SK (1986). Extraction of Schottky diode parameters from forward current-voltage characteristics. Appl. Phys. Lett..

