# Supplementary material for: Exploration of a wide bandgap semiconducting supramolecular Mg(II)-metallohydrogel derived from an aliphatic amine: a robust resistive switching framework for brain-inspired computing
Source: Sci Rep. 2023 Dec 15;13:22318. doi: 10.1038/s41598-023-48936-2 (PMC10724216; doi:10.1038/s41598-023-48936-2)
Supplement: Supplementary file 1 — Supplementary Information. [file 41598_2023_48936_MOESM1_ESM.pdf]

Supporting Information

Exploration of a Wide Bandgap Semiconducting Supramolecular Mg(II)-Metallohydrogel Derived from An Aliphatic Amine: A Robust Resistive Switching Framework for Brain-Inspired Computing

Kripasindhu Karmakar,[1,‡] Arpita Roy,[2,‡] Subhendu Dhibar,*[1] Shantanu Majumder,[2] Subham Bhattacharjee,[3] SK. Mehebub Rahaman,[1] Ratnakar Saha,[4] Priyajit Chatterjee,[5] Soumya Jyoti Ray,*[2] and Bidyut Saha*[1]

[1]Colloid Chemistry Laboratory, Department of Chemistry, The University of Burdwan, Golapbag, Burdwan-713104, West Bengal, India

[2]Department of Physics, Indian Institute of Technology Patna, Bihar-801106, India

[3]Department of Chemistry, Kazi Nazrul University, Asansol-713303, West Bengal, India

[4]School of Chemical Sciences, National Institute of Science Education and Research (NISER), Bhubaneswar, Odisha-752050, India

[5]University Science Instrumentation Centre, The University of Burdwan, Golapbag, Burdwan-713104, West Bengal, India

*E-mail: *sdhibar@scholar.buruniv.ac.in* (S Dhibar); *bsaha@chem.buruniv.ac.in* (B. Saha); *ray@iitp.ac.in* (S. J. Ray).

I. Characteristics of ITO/Mg@TMA/Cu based RRAM device with multiple cycles:

We have measured the complete IV curves for device (ITO/Mg@TMA/Cu) for consecuitive cycles and observed some variability in the multiple cycles (~ 500) as shown in Figure S1 which gets stabilised later. The initial variation can arise due to ion migration. Cu ion migration plays an important role in the formation of conductive filament for switching process. RRAM devices often depend on the movement of oxygen vacancies within the material to achieve resistive switching. However, with repeated cycling, the distribution and concentration of oxygen vacancies can change. This redistribution can alter the resistive states and affect the hysteresis behaviour observed in the I-V curve.

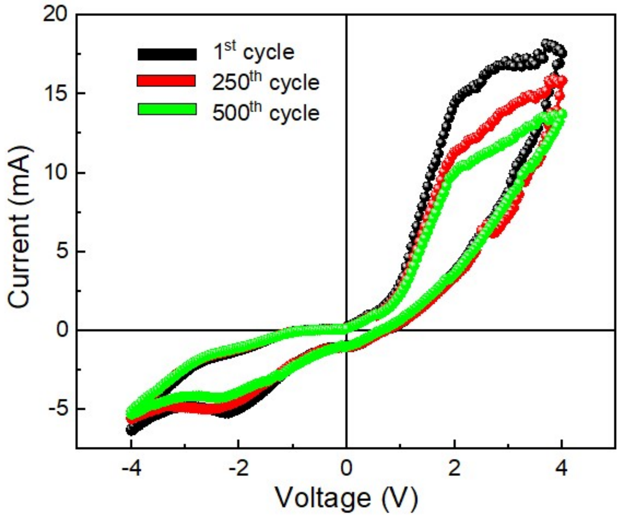

Figure S1. I-V characteristics for glass/ITO/Mg@TMA/Cu based device upto 500th cycle.

II. Characteristics of Cu/Mg@TMA/Cu based RRAM device with multiple cycles:

We have also measured the complete IV curves for device (Cu/Mg@TMA/Cu) for consecuitive cycles and observed some variability in the multiple cycles (~ 500) as shown in Figure S2 which gets stabilised later. The initial variation can arise due to ion migration. Cu ion migration and oxygen vacancy play an essential role in the formation of conductive filament for switching process.

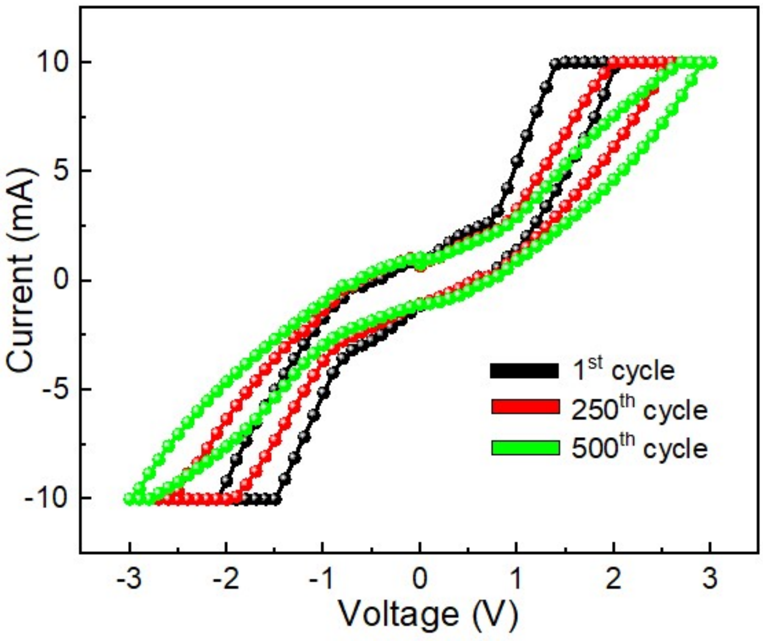

Figure S2. I-V characteristics for glass/Cu/Mg@TMA/Cu based device upto 500th cycle.

III. Filament formation using TEM and EDAX analysis:

We have measured IV characteristics for both devices with change in area between two electrodes (shown in Figure S3). But there are no more differences in hysteresis loop for changing area between two electrodes. We can confirm that there is formation of conduction filament which arise due to Cu ion migration. Cu ion migration plays an important role for resistive switching mechanism. We have also observed ion migration from TEM and EDAX analysis (shown in Figure S4((a)-(d))). From image, we observed the rod like structure after switching which confirms there is presence of filament during switching. From EDAX analysis, we can confirm absolutely that there is formation of Cu filament. There are 57 wt% Cu ions present in the sample after switching. In this way it provides a complete switching mechanism based on conductive filament model.

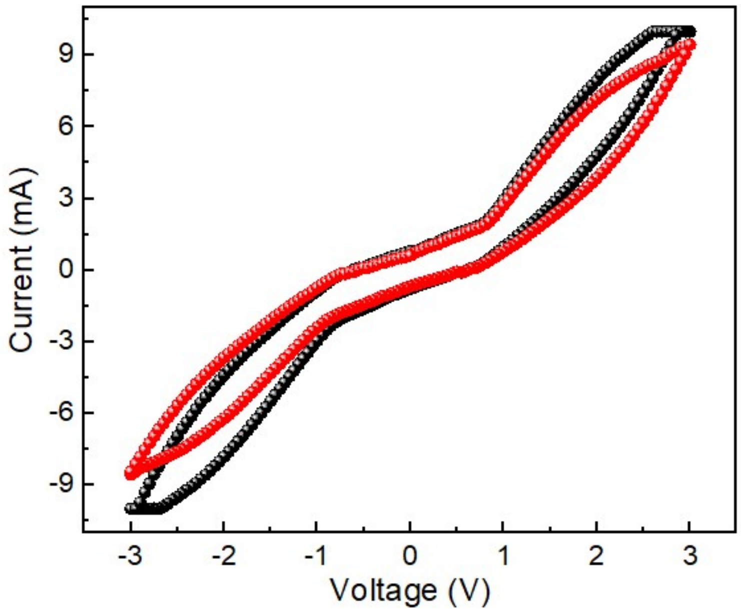

Figure S3. I-V characteristics for glass/Cu/Mg@TMA/Cu based device for changing area between two electrodes.

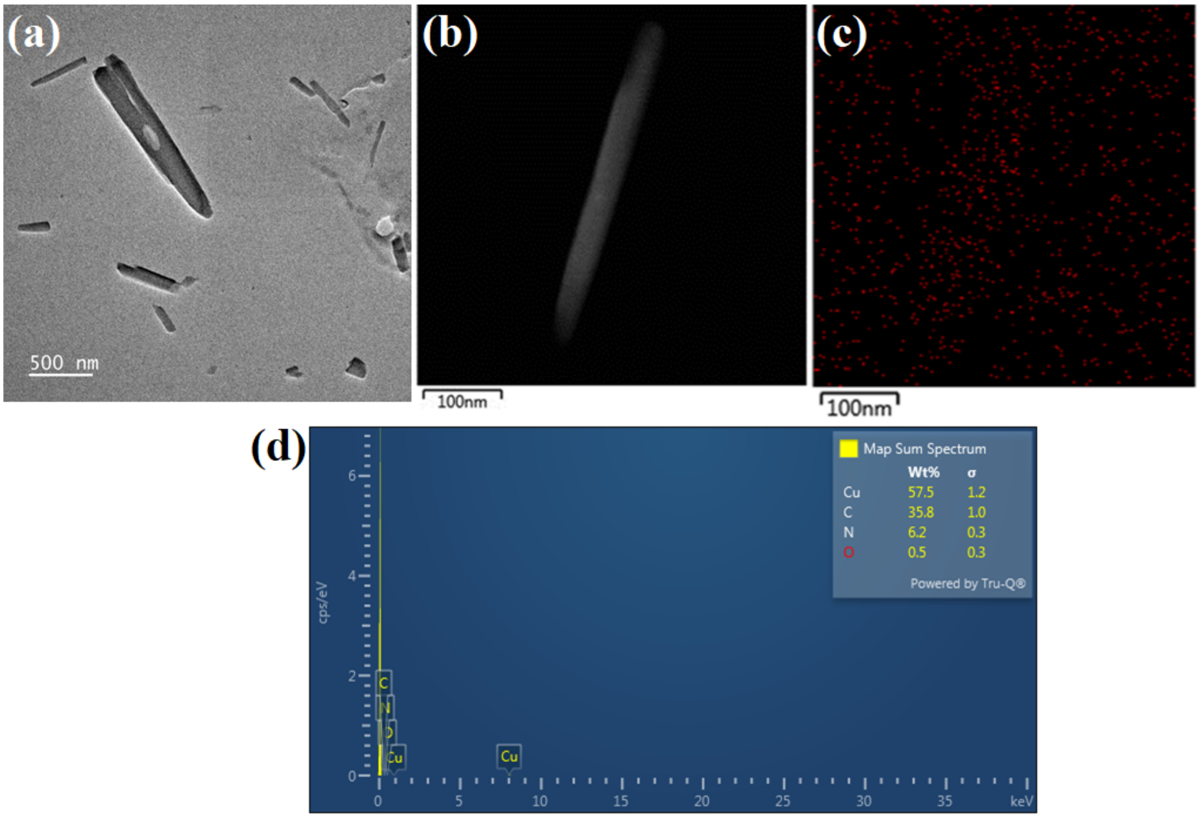

Figure S4. (a) TEM image of formation of Cu filament; (b-d) Elemental mapping of metallohydrogel which confirms the presence of Cu filament of 57 wt%.

IV. Endurance test of glass/ITO/Mg@TMA/Cu based device:

To better understand the consistency of the switching process, we also performed an endurance test over 5000 switching cycles consecutively at room temperature for the device (ITO/Mg@TMA/Cu), as shown in Figure S5. The switching procedure for this device is reliable for up to 5000 switching cycles respectively. The endurance test shows that the switching behaviour is robust because the average ON/OFF ratio is around 60 for this device. It implies that this device can continue to function as intended in terms of memory response over an extended period without experiencing any degradation.

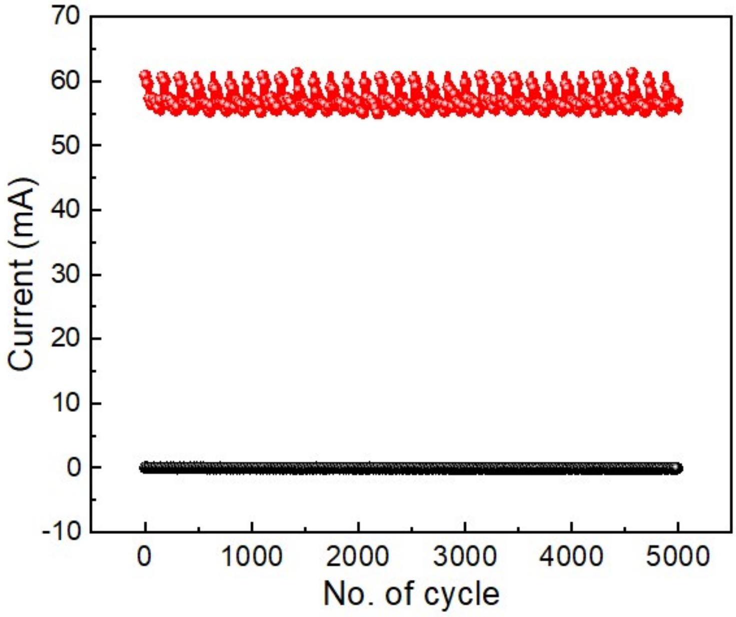

Figure S5. Endurance test of glass/ITO/Mg@TMA/Cu based device.

V. Retention test of glass/ITO/Mg@TMA/Cu based device:

We have also performed retention test upto 10^3 sec for the device (ITO/Mg@TMA/Cu) at room temperature as shown in Figure S6. The switching procedure for this device is stable up to 10^3 sec. Here, we have observed that for this device ON/OFF ratio is around 100. So, we can conclude that this device can retain data upto 10^3 sec without any degradation.

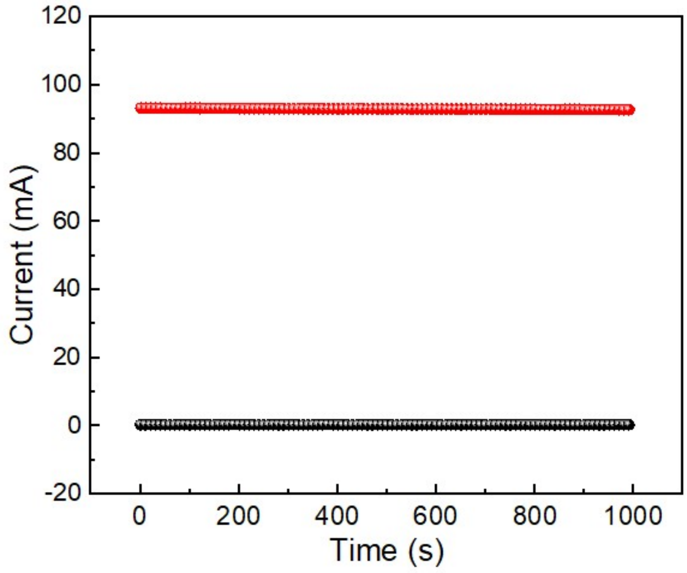

Figure S6. Retention test of glass/ITO/Mg@TMA/Cu based device.
